# RNA sequencing analysis of *Cymbidium goeringii* identifies floral scent biosynthesis related genes

**DOI:** 10.1186/s12870-019-1940-6

**Published:** 2019-08-02

**Authors:** Mummadireddy Ramya, Pue Hee Park, Yu-Chen Chuang, Oh Keun Kwon, Hye Ryun An, Pil Man Park, Yun Su Baek, Byoung-Chorl Kang, Wen-Chieh Tsai, Hong-Hwa Chen

**Affiliations:** 1 Floriculture Research Division, National Institute of Horticultural & Herbal Science (NIHHS), Rural Development Administration (RDA), Wanju, 55365 South Korea; 2 Department of Life Sciences, National Cheng Kung University (NCKU), Tainan, 701 Taiwan; 3 Department of Horticultural Science and Biotechnology, Seoul National University (SNU), Seoul, 08826 South Korea; 4 Institute of Tropical Plant Sciences, National Cheng Kung University, Tainan, 701 Taiwan; 5 Orchid Research and Development Center, National Cheng Kung University, Tainan, 701 Taiwan

**Keywords:** *Cymbidium goeringii*, Floral scent, Transcriptome, RNA-seq, Sesquiterpenes

## Abstract

**Background:**

*Cymbidium goeringii* belongs to the Orchidaceae, which is one of the most abundant angiosperm families. *Cymbidium goeringii* consist with high economic value and characteristics include fragrance and multiple flower colors. Floral scent is one of the important strategies for ensuring fertilization. However, limited genetic data is available in this non-model plant, and little known about the molecular mechanism responsible for floral scent in this orchid. Transcriptome and expression profiling data are needed to identify genes and better understand the biological mechanisms of floral scents in this species. Present transcriptomic data provides basic information on the genes and enzymes related to and pathways involved in flower secondary metabolism in this plant.

**Results:**

In this study, RNA sequencing analyses were performed to identify changes in gene expression and biological pathways related scent metabolism. Three cDNA libraries were obtained from three developmental floral stages: closed bud, half flowering stage and full flowering stage. Using Illumina technique 159,616,374 clean reads were obtained and were assembled into 85,868 final unigenes (average length 1194 nt), 33.85% of which were annotated in the NCBI non redundant protein database. Among this unigenes 36,082 were assigned to gene ontology and 23,164 were combined with COG groups. Total 33,417 unigenes were assigned in 127 pathways according to the Kyoto Encyclopedia of Genes and Genomes pathway database. According these transcriptomic data we identified number of candidates genes which differentially expressed in different developmental stages of flower related to fragrance biosynthesis. In q-RT-PCR most of the fragrance related genes highly expressed in half flowering stage.

**Conclusions:**

RNA-seq and DEG data provided comprehensive gene expression information at the transcriptional level that could be facilitate the molecular mechanisms of floral biosynthesis pathways in three developmental phase’s flowers in *Cymbidium goeringii,* moreover providing useful information for further analysis on *C*. *goeringii,* and other plants of genus *Cymbidium.*

**Electronic supplementary material:**

The online version of this article (10.1186/s12870-019-1940-6) contains supplementary material, which is available to authorized users.

## Background

Orchidaceae is one of the largest and also most widespread families of flowering plants, with more than 25,000 species [1]. The genus *Cymbidium* belongs to the Orchidaceae family [2, 3] and is economically important due to their beautiful and fragrant flowers. *Cymbidium* consists of nearly 55 species distributed, mainly in tropical and subtropical Asia and reaching as far south as Papua New Guinea and Australia [4]. *Cymbidium goeringii* (spring orchid) is a fragrant flower. It is also threatened by over-collection, habitat disturbance and fragmentation [5, 6]. *Cymbidium* has remembered its status as a celebrity orchid for thousands of years since antiquity: Asian people treasure *Cymbidium* orchid flowers because of the fragrant blooms, flamboyant flower displays (of up to 30 flowers on a single spike), and attractive flowers that come in many different colors.

New cultivars with different floral traits, such as color, morphology, and scent, have been generated mainly by classical cross-breeding and mutation breeding. Scent is an important property of flowers and plays a vital role ecologically, economically, and aesthetic properties of flowering plants. Most of the plants possess a distinct and unique floral scent. Analysis of the biosynthesis mechanisms involved in floral scent is necessary to understand the fine-scale molecular functions and to breed new cultivars through regulation of floral scent.

Floral scents are composed of various volatile organic compounds, such as terpenoids, phenylpropanoids, benzenoids, fatty-acids and their derivatives. Terpenoids belong to a large family of specialized metabolites, and their corresponding alcohols possess useful properties such as fragrance and flavor [7]. The floral scents of ornamental plants such as *Rosa hybrid* [8], tree peony [9], *Lilium spp.* [10], *Prunus mume* [11], and *Syringa oblata* [12] have been thoroughly studied. The chemical structures of many floral scent compounds have recently been described and the biosynthesis pathways have been investigated in roses [13]. Volatile terpenoids such as isoprene (C5), monoterpenes, (C10) and sesquiterpenes (C15) constitute the largest class of plant volatile compounds. Terpenoids are produced from isopentenyl diphosphate (IPP) and dimethyl allyl diphosphate (DMAPP), which are C5 carbon precursors. IPP and DMAPP are derived from two alternative biosynthetic mevalonic-acid (MVA) and 2-c-methylerythritol 4-phosphate (MEP) pathways, localized in the cytosol and plastids, respectively. The successive head-to-tail condensation of IPP and DMAPP by the action of prenyltransferases generates the direct precursors of terpenes, geranyl diphosphate (GPP), geranylgeranyl diphosphate (GGPP) in plastids, and farnesyl diphosphate (FPP) in cytosol or mitochondria. In the final steps, terpene synthases covert cytosolic FPP to sesquiterpenes and plastid terpene synthases GPP and GGPP into monoterpenes and diterpenes. Most of the terpenoid biosynthesis related enzymes [e.g., 1-deoxy-d-xylulose-5-phosphate synthase **(**DXS), 3-hydroxy-3-methylglutaryl-CoA synthase (HMGR), 1-deoxy-d-xylulose-5-phosphate reductoisomerase (DXR), phosphomevalonate kinase (PMK), 4-(cytidine 5′-diphospho)-2-C- linalool synthase (LIS), 1-hydroxy-2-methyl-2-(E)-butenyl-4-diphosphate reductase (HDR), and acetoacetyl-CoA transferase (AACT), have been analyzed and expressed at various *Hedychium coronarium* floral developmental stages [14–17]. However, biosynthetic pathways and regulatory mechanisms of floral scent in Oncidium orchid plants are largely unknown.

Compared to other orchids, very little genomic data are available on the regulatory mechanisms of floral scent biosynthesis in *Cymbidium goeringii,* making it difficult to further study the molecular basis of floral fragrance. In recent years, RNA-Seq based on Illumina sequencing techniques has provided attractive opportunities to dramatically improve the efficiency of gene discovery. RNA-Seq coupled with digital gene expression (DGE) profiling have been used for studying flowers in many ornamental plants including *Syringa oblata* [18]*, Chimonanthus praecox* [19], *Cymbidium sinense* [20], *Cymbidium ensifolium* [21], *Salvia splendens* [22]. Genes involved in floral scent pathways, flowering time, signal transduction, and developmental of floral structure were studied.

In this study, we generate the transcriptome of *Cymbidium goeringii* flowers at various developmental stages using RNA-Seq, and digital gene expression using Illumina technology. We examined differentially expressed genes (DEG) with the “Fragments per Kilobase of Transcript per Million Fragments Mapped” (FPKM) method [23, 24]. The comprehensive gene expression information at the genomic level facilitated our understanding of the molecular mechanisms underlying *C*. *goeringii* floral scent. Our results provide an important resource for further investigating flowering pathways and other biological pathways in other orchid species.

## Results

### Changes in the volatiles during the flower development of *C. goeringii*

**Fig. 1:**
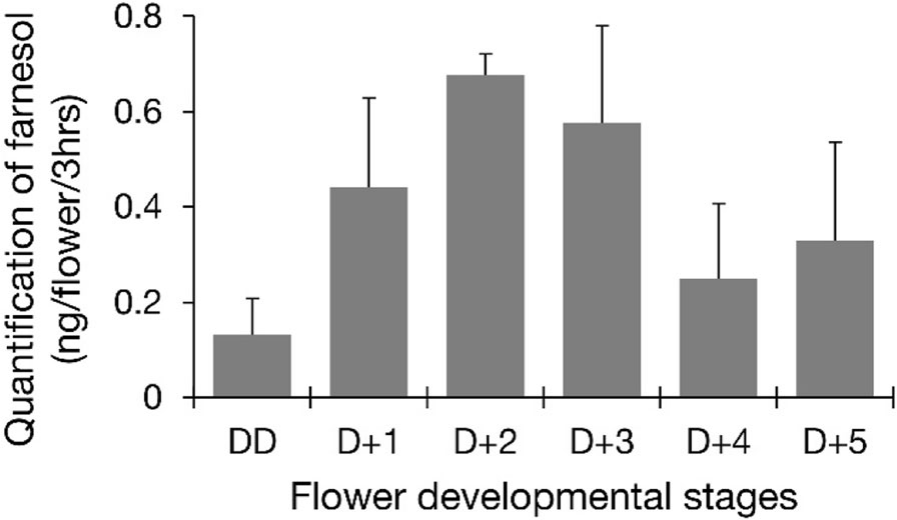
Changes in the floral volatile profiles during flower development in *C. goeringii*. The emitting patterns of franesol from the anthesis day (DD) to day 5 post anthesis (D + 5) in *C. goeringii*. Data are mean ± SE of triplicate measurements

The dominant floral volatile organic compounds of *C. goeringii* were identified as farnesol, methyl epi-jasmonate, (E)-β-farrnesene, and nerolidol (Table 1). Among them sesqiterpenes are the major compounds in scent profile. In addition we analyzed the changes of main floral volatile farnesol from anthesis day (DD) to day 5 post anthesis (D + 5). Farnesol had highest emission on D + 2 stage compared to other compounds (Fig. 1). Most of these floral volatile compounds belong to terpenoids, and were considered to be generated through the terpenoid pathway in *C. goeringii*.

**Table 1:** Major volatile components of *C. goeringii* flowers

Retention Time	Area (%)	Compound
19.05	1.46	(E)-β-Famesene
21.47	0.76	Nerolidol
23.82	4.8	Methyl epi-jasmonate
24.7	38.76	Farnesol

### Transcriptome sequencing of *C. goeringii* flowers

**Fig. 2:**
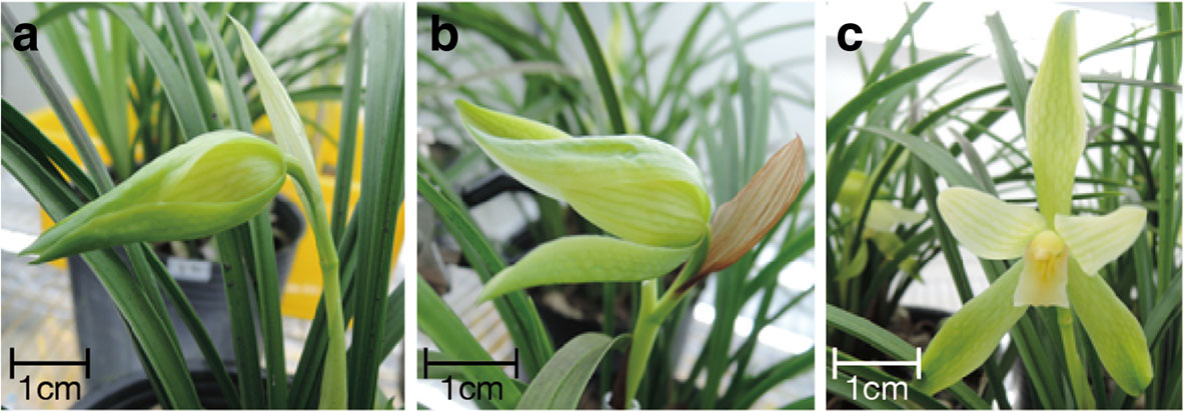
Three flower developmental stages of *C. goeringii* for transcriptome construction. Pictures of *C. goeringii* flowers: a flower at the bud stage (**a**), a flower at the half flowering stage (**b**), and a flower at the full flowering stage (**c**) Scale bar =1 cm

To study the molecular basis of the scent biosynthesis in *C. goeringii*, flowers at the bud stage (A), the half flowering stage (B), and the full flowering stage (C) were chosen to construct three cDNA libraries (Fig. 2), which represented the onset, increase and peak phase of the scent emission pattern in *C. goeringii* (Fig. 1). A total of 162 M raw reads were obtained, and 54.44 M, 53.95 M, and 51.22 M clean reads were then generated for each developmental stage after separating out low-quality reads, respectively (Table 2). As the genomic sequences for *Cymbidium* family were unavailable, de novo assembly approach was applied by using Trinity software. A total of 85,868 unigenes were thus yielded with an average length of 1194-bp and N50 of 1880-bp. Among them, Trinity defined 42,629 unigenes as alternatively spliced transcripts, which included 10,609 gene clusters with 2 to 76 isoforms for each one. The other 43,239 unigenes were considered as distinct transcripts from single genes. The length distribution of all unigenes was then analyze and showed that 38.9% unigenes had the length between 1000-bp and 3000-bp, followed by 34.7% unigenes in the range of 300-bp to 1000-bp, and 20% unigenes within 300-bp. (Additional file 1: Figure S1a). The transcript abundance of each individual unigenes in the *C. goeringii* floral transcriptome was represented by the log2 of FPKM values (Fig. 4a).

**Table 2:** Summary of the *C. goeringii* transcriptome

Sample	A	B	C	Total
Total Raw Reads	55,086,718	54,689,708	51,987,104	161,763,530
Total Clean Reads	54,443,430	53,950,030	51,222,914	159,616,374
Q20 (%)	97.88%	97.86%	97.79%	–
N (%)	0.00%	0.00%	0.00%	–
GC (%)	45.98%	47.03%	46.51%	–
Number of unigenes	85,868			
Mean Length (bp)	1,194			
N50 (bp)	1,880			

### Functional annotations of all unigenes

The putative function of the unigenes in the *C*. *goeringii* floral transcriptome was annotated by searching against the public databases using BLASTX or BLASTN with E-value cutoff 10^− 5^, including NCBI non-redundant (NR) and non-redundant nucleotide (NT) database, Swiss-Prot protein database, Clusters of Orthologous Groups of proteins (COG), Kyoto Encyclopedia of Genes and Genomes (KEGG), and Gene Ontology (GO). A total of 56,808 unigenes (66.2%) were annotated with a function by using this strategy. For each database, 63.6% unigenes were matched to the proteins in NR database, followed by 51.7% in NT database, 42% in GO database, 43% in Swiss-Prot database, 38.9% in KEGG database, and 27.5% in COG database (Table 3). Among the remaining unannotated unigenes (33.8%), 47.7% of them had the length within 300-bp (Additional file 1: Figure S1b), which indicated that they were too short to contain the conserved region. Other unannotated unigenes, especially those with long size (larger than 500-bp, 22.9%), might be specific to *C*. *goeringii.*

**Table 3:** Summary of annotations on the unigenes in the *C. goeringii* floral transcriptome against public databases

Database	NR	NT	Swiss-Prot	COG	KEGG	GO	All	Total Unigenes
Number of annotated unigenes	54,640	44,403	36,911	23,614	33,417	36,082	56,808	85,868
Proportion	63.6%	51.7%	43.0%	27.5%	38.9%	42.0%	66.2%	100.0%

The annotation results against the NR database were next applied to analyze the E-value distribution and species specificity. Among the unigenes with significant hits, 17.9% unigenes showed exact match (E-value = 0), followed by 22.1% with very strong homology (0 < E-value ≤1.0e^− 100^), 24.6% with strong homology (1.0e^− 100^ < E-value ≤1.0e^− 45^), and remaining 35.4% with moderate homology (E-value > 1.0e^− 45^) (Additional file 2: Figure S2a). In addition, there were a total of 562 plant species contributing to the annotated unigenes. Intriguingly, we found that 19.9% unigenes showed top hits to the proteins from *Vitis vinifera*, followed by *Theobroma cacao* (6.8%), *Setaria italic* (5.8%), and *Oryza sativa* (5.2%) (Additional file 2: Figure S2b).

### GO and COG categorization of unigenes

**Fig. 3:**
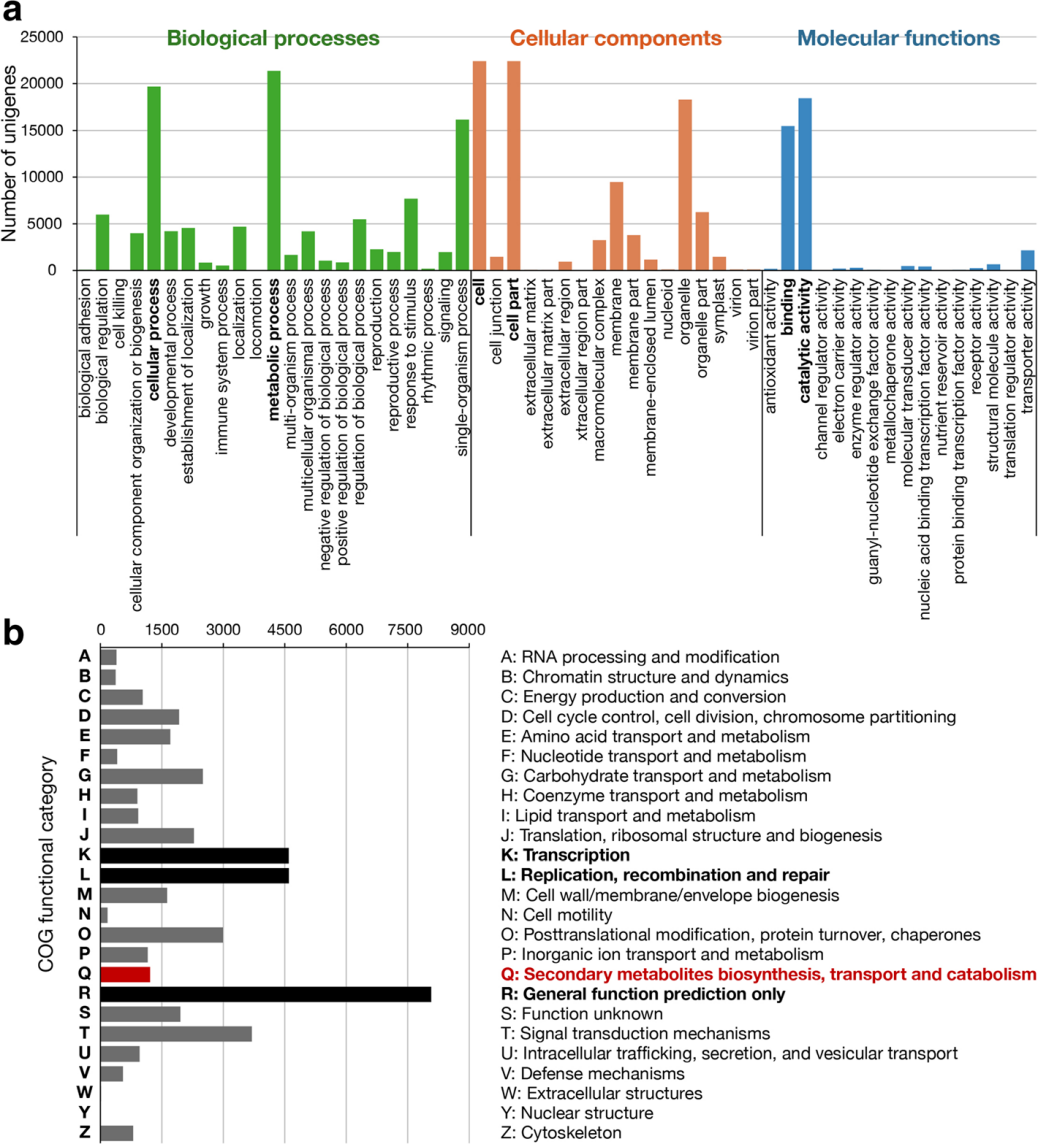
Functional characterization of the *C. goeringii* floral transcriptome. **a** GO assignments. **b** COG classification. The reprentative category/subcatefory was shown in bold, and the one denoted as “secondary metabolites biosynthesis” was shown in red

The functional categorization of the *C*. *goeringii* floral transcriptome were performed by analyzing the BLAST results against GO and COG databases. A total of 36,082 unigenes with at least a GO term were assigned to three main GO categories, including “biological processes”, “cellular components” and “molecular functions”, and further 56 subcategories. The representative subcategories in “biological processes” were “metabolic process” (59.2%) and “cellular process” (54.5%), the top two subcategories in “cellular components” were “cell” (62.0%) and “cell part” (62.0%), while the ones in “molecular functions” were “binding” (42.9%) and “catalytic activity” (51.1%) (Fig. 3a), which implied the enriched metabolism and biochemical process during flower developmental stages in *C*. *goeringii*.

For COG categorization, 23,614 unigenes were divided into 25 COG categories. Some of the unigenes were assigned to more than one category. The largest proportion of unigenes belonged to the undefined functional “general function prediction only” (18%), followed by “transcription” (10.3%) and “replication, recombination and repair” (10.3%), and “signal transduction mechanisms” (8.2%). In particular, our interest “secondary metabolites biosynthesis, transport and catabolism” category accounted for 2.7% of total annotated unigenes by COG Fig. 3b), which were to further study on their role in the floral scent biosynthesis pathway in *C*. *goeringii*.

### Terpene biosynthesis

The floral volatile compounds in *C*. *goeringii* belonged to the terpenoid class, leading us to analyze the KEGG annotation results regrading terpenoid biosynthesis. A total of 197 unigenes annotated with the pathway ID ko00900, which indicates “terpenoid backbone biosynthesis”, were isolated, and 70 genes were then confirmed by local BLAST. These unigenes were further assigned to two distinct pathways according to the sequence homology, including 32 unigenes in the cytosolic MVA pathway and 38 unigenes in the plastidial MEP pathway. The E-value for these unigenes with their homology proteins and their expression levels was shown in Additional file 3: Table S1 and Additional file 4: Table S2.

**Fig. 4:**
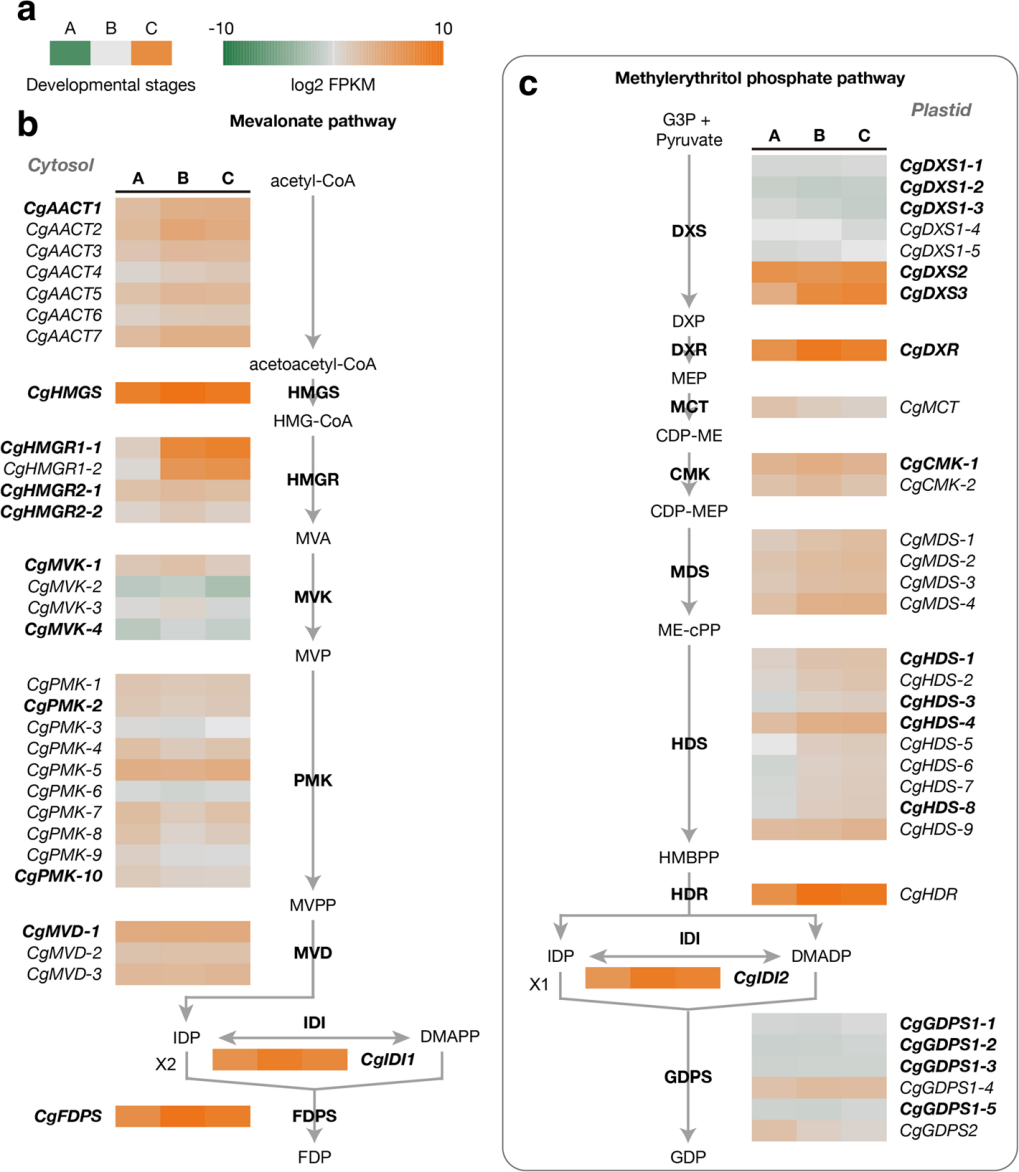
The expression profiles of putative genes encoding enzymes involved in terpene backbone biosynthesis. **a** The three squares indicate the gene expression levels (log2 FPKM) in *C. goeringii* flowers on the stage of A, B, and C, which are shown by a color gradient from orange to grey to green. **b** and **c** Expression levels of putative genes in the cytosol mevalonate (MVA) pathway (**b**) and plastidial methylerythritol phosphate (MEP) pathway (**c**). The abbreviated names of enzymes in each catalytic step are in bold. The putative unigenes contained the full-length open reading frames (ORFs) are shown in bold. The intermediate compounds in both pathways are listed by alphabetical order: CDP-ME, 4-diphosphocytidyl-2-C-methylerythritol; CDP-MEP, 4-diphosphocytidyl-2-C-methyl-D-erythritol 2-phosphate; DXP, 1-deoxy-D-xylulose 5-phosphate; G3P, glyceraldehyde-3-phosphate; HMBPP, 4-hydroxy-3-methyl-but-2-enyl pyrophosphate; HMG-CoA, *S*-3-hydroxy-3-methylglutaryl-CoA; MVP, mevalonate-5-phosphate; MVPP, mevalonatediphosphate; ME-cPP, 2-C-methyl-D-erythritol 2,4-cyclodiphosphate

In the MVA pathway (Fig. 4b), seven unigenes were identified as acetyl-CoA C-acetyltransferase (AACT), while only one unigene was annotated as hydroxymethylglutaryl-CoA synthase (HMGS). Four unigenes were found as hydroxymethylglutaryl-CoA reductase (HMGR), which contained two clusters with two isoforms for each one. A total of four, ten, three gene clusters was identified as mevalonate kinase (MVK), phosphomevalonate kinase (PMK), and diphosphomevalonate decarboxylase (MVD), respectively. Interestingly, two unigenes were annotated as isopentenyl-diphosphate delta-isomerase (IDI). Subcellular localization analysis by using TargetP (http://www.cbs.dtu.dk/services/TargetP/) assigned the one without signal peptide to the MVA pathway (*CgIDI1*), and the other with chloroplast transit peptide to the MEP pathway (*CgIDI2*).

For the MEP pathway (Fig. 4c), seven unigenes were identified as 1-deoxy-D-xylulose-5-phosphate synthase (DXS), and five of them belonged to one gene cluster. The following 1-deoxy-D-xylulose-5-phosphate reductoisomerase (DXR) and 2-C-methyl-D-erythritol 4-phosphate cytidylyltransferase (MCT) were represented by one gene each. A total of two, four, six gene clusters was identified as 4-diphosphocytidyl-2-C-methyl-D-erythritol kinase (CMK), 2-C-methyl-D-erythritol 2,4-cyclodiphosphate synthase (MDS), and (E)-4-hydroxy-3-methylbut-2-enyl-diphosphate synthase (HDS), respectively. One unigene was annotated as 4-hydroxy-3-methylbut-2-enyl diphosphate reductase (HDR).

The second step of terpene biosynthesis is the condensation of IDP and DMADP catalyzed by a group of short-chain prenyltransferases to produce prenyl diphosphates, the precursors of all terpenes. Here, we focused on farnesyl diphosphate synthase (FDPS) generating FDP for sesquiterpene synthesis. The KEGG annotation results represented one and six unigenes encoding FDPS and GDPS, respectively (Fig. 2b, c). For GDPS, five unigenes of them belonged to one gene cluster.

**Fig. 5:**
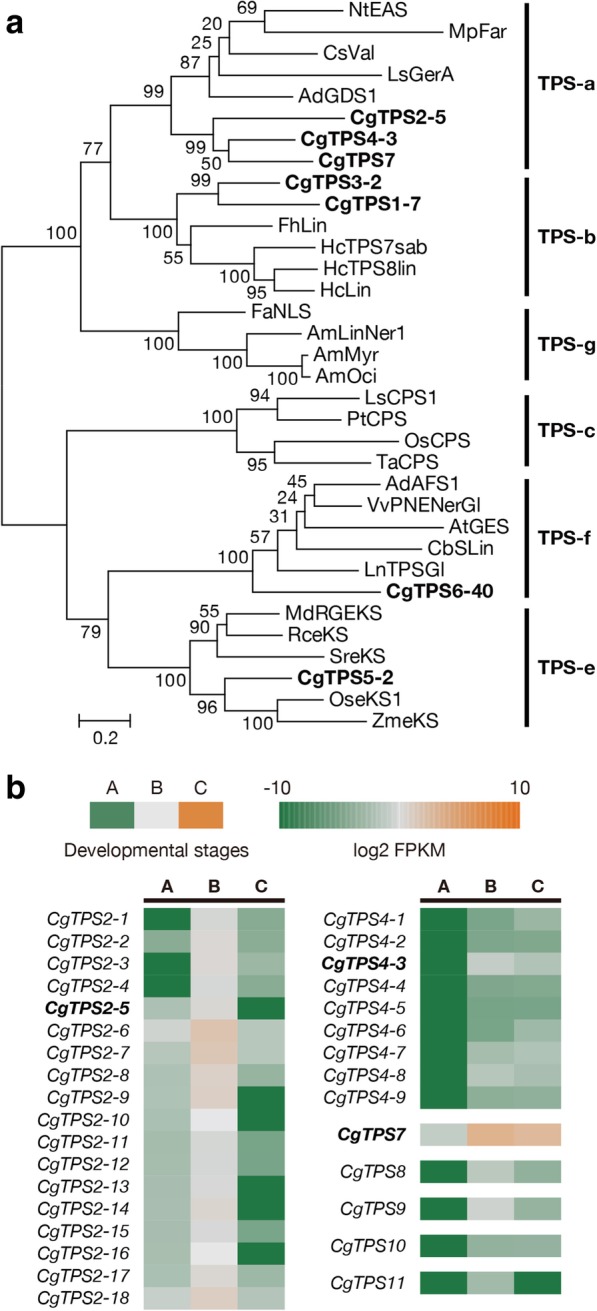
Classification and expression profiles of the putative terpene synthases (TPS) genes. **a** Phylogenetic analysis of the subfamilies of putative TPSs with TPSs identified from other species. TPSs identified from *C. goeringii* floral transcriptome are shown in bold. Bootstrap values were calculated as a percentage of 1000 replicates. The scale bar represents 0.2 substitutions per site. **b** The expression profiles of unigenes encoding enzymes in TPS-a family. The putative unigenes contained the full-length ORFs are shown in bold

In the last step, terpene synthase (TPS) catalyzes the production of terpene by using prenyl diphosphates as substrates. In order to identify TPS in the *C*. *goeringii* floral transcriptome, the KEGG annotation results with the pathway ID ko00902, ko00909, indicating “monoterpenoid biosynthesis”, and “sesquiterpenoid and triterpenoid biosynthesis”, respectively, were isolated. In addition, putative unigenes encoding TPS in the transcriptome were also explored by using tBLASTX against the TPS sequences collected from other plants (E-value < 1.0e^− 5^). The combination of both results yielded a total of 169 unigenes as TPSs. However, sequence analysis showed only a small number of them containing the full-length open reading frames (ORFs) (*N* = 7), while the most ones were shorter alternative spliced forms. Phylogenetic analysis classified the seven TPSs into TPS-a, TPS-b, and TPS-e/f families (Fig. 5a). Enzymes in TPS-a group are usually characterized as sesquiterpene synthases (STPs). In addition to the three unigenes grouped into TPS-a family, four unigenes were also annotated as STPSs (Fig. 5b), although they did not contain full-length ORFs. The expression profiles of these seven unigenes and their gene clusters was exhibited in Fig. 5. Also, their expression levels were shown in Additional file 5: Table S3. Among them, *CgTPS7* had the highest gene expression levels and was considered to play important role in the sesquiterpene biosynthesis in *C*. *goeringii* flowers.

### Analysis of differential expressed genes at three flloral developmental stages

Following by the annotation analysis, differentially expressed genes (DEGs) were next determined at three flower developmental stages. The paired analysis between the stages was performed with the criteria of a false discovery rate below 0.001 and two-fold-change. A total of 21,620 and 15,815 DEGs were thus isolated in A vs B and A vs C, respectively (Table 4), which suggests profound changes in gene expression profiles at the flower opening. Among all DEGs, the number of the down-regulated unigenes was accounted for a significant proportion (> 60% in both comparisons), however, the number of the up-regulated unigenes was still increased along with the development proceeding (Table 4), which was concomitant with the rise of the scent volatiles.

**Table 4:** The number of DEGs in the *C. goeringii* floral transcriptome

DEGs Set	A vs B			A vs C	
	All DEGs	KEGG DEGs		All DEGs	KEGG DEGs
All DEGs	21,620	6,001		15,815	5426
Up-regulated	5000 (23.1%)	2044 (34.1%)		5952 (37.6%)	2235 (41.2%)
Down-regulated	16620 (76.9%)	3957 (65.9%)		9863 (62.4%)	3191 (58.5%)

**Fig. 6:**
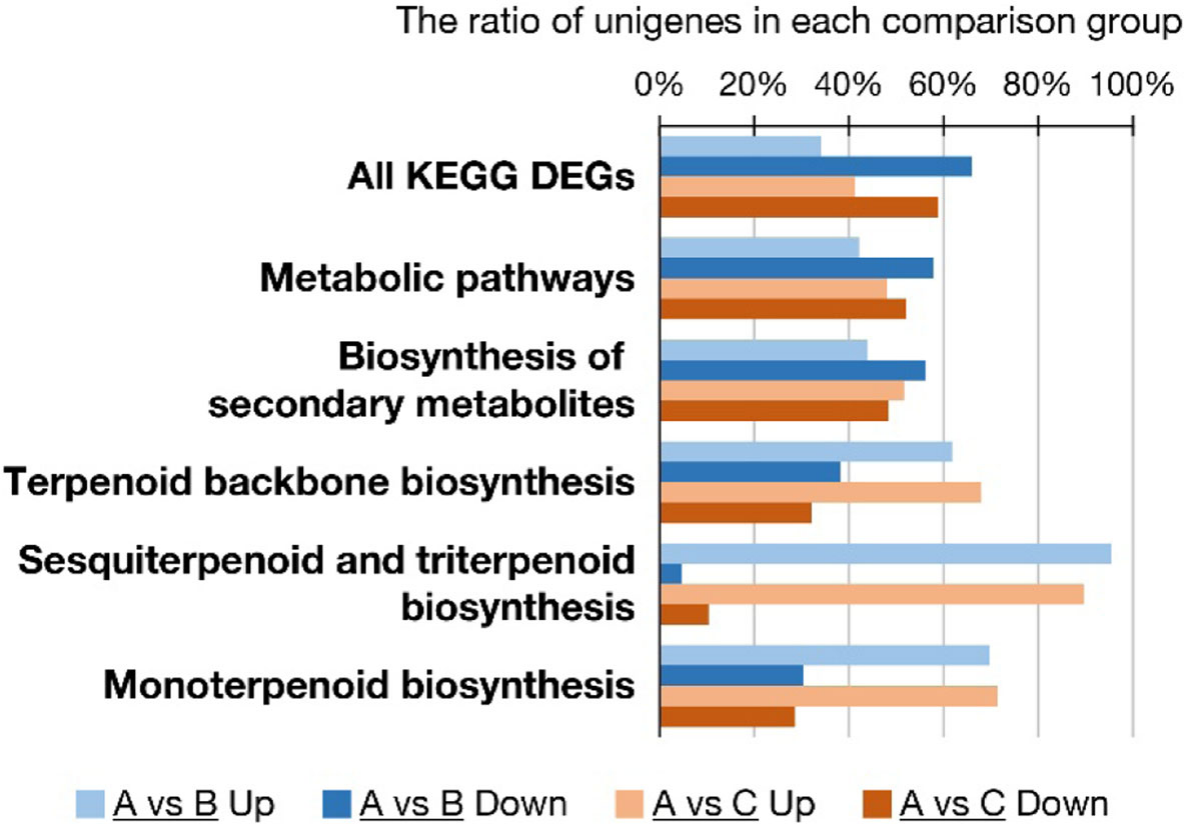
The DEGs in the KEGG pathways regarding terpene biosynthesis. The ratio of the up-regulated (light color) or down-regulated (dark color) DEGs compared to total DEGs in each category. The DEGs in A vs B comparison groups were shown in blue, while those in A vs C were in orange

Further analysis of these DEGs by using KEGG annotation showed that the ratio of up-regulated unigenes to down-regulated ones in both comparison sets was similar to that of total unigenes. However, in the top two enriched pathways, “metabolic pathways” and “biosynthesis of secondary metabolites” (Additional file 6: Table S4), the number of up-regulated DEGs was accounted for a greater proportion (Fig. 6, ~ 40% in A vs B, and ~ 50% in A vs C). Furthermore, in the pathways regarding terpene biosynthesis, the number of up-regulated DEGs was even much higher than that of down-regulated ones (Fig. 6). The approximately 90% of up-regulated DEGs in “sesquiterpenoid and triterpenoid biosynthesis” pathway was also consistent with the large amounts of sesquiterpeoids detected in *C*. *goeringii* flowers.

### Identification of candidate transcription factors for regulating terpene biosynthesis

Recently, a growing number of studies have reported that several types of transcription factors (TFs) are involved in the regulation for terpene biosynthesis, including basic helix-loop-helix (bHLH), basic leucine zipper (bZIP), ethylene response factor (ERF), NAC, MYB, and WRKY. Here, a total of 2,307 TFs were identified in the *C*. *goeringii* floral transcriptome by a search to iTAK (E-value ≤1.0e^− 5^) and confirmed by local BLAST, which contained 456 gene clusters and 723 distinct unigenes. These 1,179 gene groups were classified into 64 putative TF familys, with the three largest ones being bHLH (73), ERF (71), and C2H2 zinc finger proteins (65) (Additional file 7: Figure S3).

**Fig. 7:**
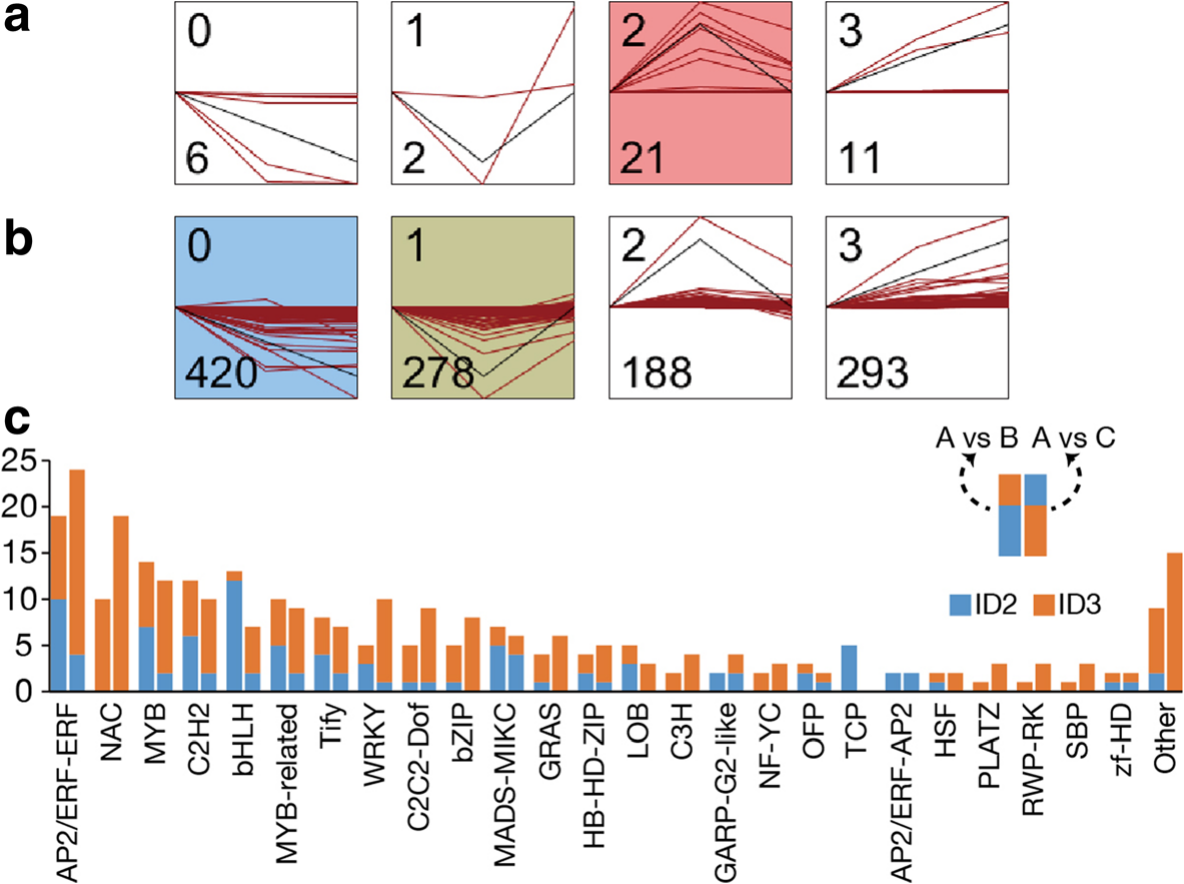
The clustering analysis of putative enzyme genes and candidate upstream transcription factors. The STEM software clustered the expression pattern of putative enzyme genes (**a**) and candidate upstream TFs (**b**) into four individual profiles. The profiles in color indicate statistical significance (*P* < 0.01). The number on the upper left corner of every profile is the profile ID, and the one on the lower left corner is the number of unigenes identified. The red lines show individual gene expression pattern and the black lines indicate the model expression profiles. The x-axis shows the three-time points (A, B, and C). **c** The DEG analysis of TFs in profile ID 2 (in blue) and ID 3 (in orange). The left column indicates DEGs isolated form A vs B and the right one is for A vs C

To isolate candidate TFs for regulating terpene biosynthesis, we first analyzed the expression pattern of the structural genes encoding putative enzymes involved in terpene biosynthesis. The clustering analysis was performed by using the Short Time-series Expression Miner (STEM) software based on their FPKM values at three floral stages [25], and four distint profile was generated for 40 putative enzyme genes (Fig. 7a). Most putative enzyme genes were classified into STEM profile ID 2 (*N* = 21) and 3 (*N* = 11), corresponding to the peaked expression on B and C, respectively. We hypothesize that the candidate TFs should exhibit expression patterns concomitant to that of the putative enzyme genes. Therefore, among the four distint profile generated by STEM (Fig. 7b), we selected STEM profile ID 2 (*N* = 188) and 3 (*N* = 293), which also showed peak expression on B and C, respectively. Further DEG analysis in these 481 TFs showed that there were 153 up-regulated DEGs in A vs B and 178 ones in A vs C. Intriguingly, we found that the known TFs for regulating terpene biosynthesis, including ERF, NAC, MYB, and bHLH, occupied a large proportion in these DEGs.

**Fig. 8:**
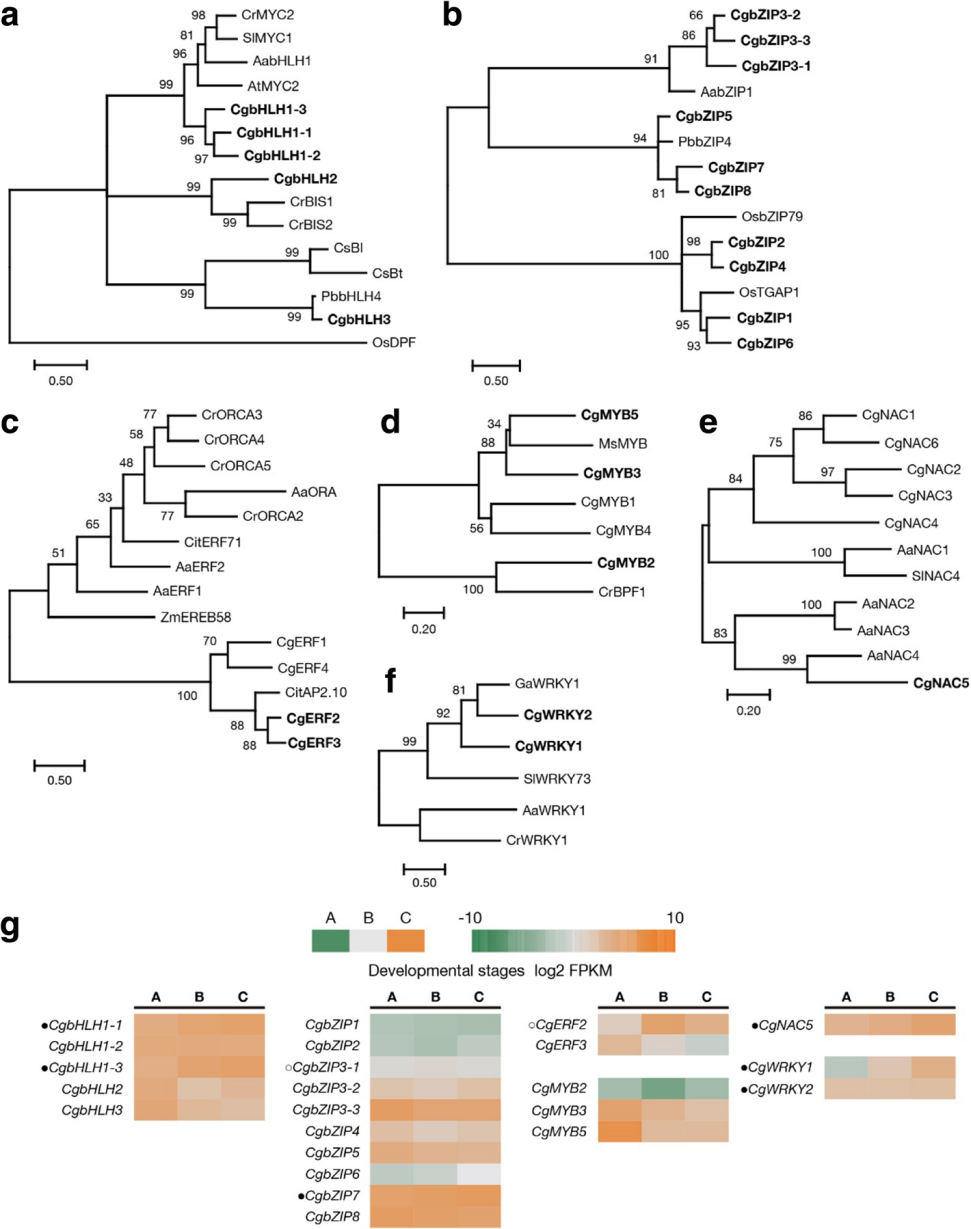
Identification of candidate transcription factors regulating terpenoids. Phylogenetic tree inferred from the amino sequences of the TFs regulating terpenoids in other species with their close relatives in *C. goeringii*. Various types of TFs were shown: bHLH (**a**), bZIP (**b**), ERF (**c**), MYB (**d**), NAC (**e**), WRKY (**f**). The phylogenetic tree was constructed either with the maximum-likelihood (a, b, c, e, f) or neighbor-joining method (**d**). Bootstrap values were calculated as a percentage of 1000 replicates. The closest candidate TFs with the TFs regulating terpenoids were shown in bold. **g** Expression levels of the candidate TFs shown in bold in (**a**) to (**f**). The candidate TFs classified into STEM profile ID 2 and 3 were labeled with an open circle and a dark dot, respectively

Therefore, we next isolated the candidate TFs by using tBLASTX against the TFs regulating terpenoids in other species (E-value < 1.0e^− 50^), which are summarized [26] (Additional file 7: Table S5). Phylogenetic analysis was then applied to identify the close relatives with the TFs regulating terpenoids (Fig. 8a, Additional file 8: Table S5). Among these TFs, several of them indeed showed concomitant with the expression patterns of the putative enzyme genes (i.e. STEM profile ID 2 and 3) (Fig. 8b, Additional file 8: Table S5). These included *CgbHLH1* and *CgbZIP3*, homologus genes of AabHLH1 and AabZIP1 regulating artemisinin biosynthesis in *Artemisia annua*, respectively [27, 28], *CgbZIP7*, a homologus gene of PbbZIP4 regulating monoterpene biosynthesis in *Phalaenospis bellina* [29], *CgERF2*, a homologus gene of CitAP2.10 associating with sesquiterpene (+)-valencene synthesis in sweet orange [30], *CgNAC5*, a homologus gene of AaNAC4 regulating monoterpene synthesis in kiwifruit [31], and *CgWRKY1* and *CgWRKY2*, homologus genes of GaWRKY1 regulating sesquiterpene (+)-δ-cadinene synthesis in cotton [32]. The possibility of these candidate TFs involved in the regulation of terpene biosynthesis in *C*. *goeringii* floral transcriptome was worth further study.

### qRT-PCR analysis

**Fig. 9:**
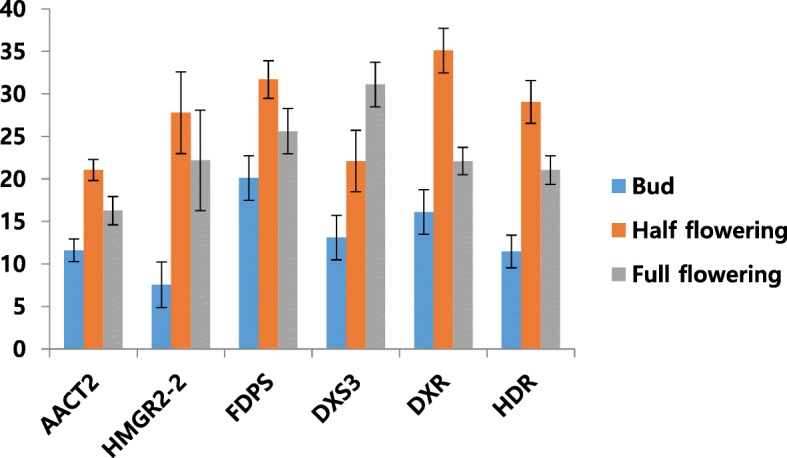
The expression analysis of putative terpenoid biosynthesis genes by qRT-PCR in *Cymbidium goeringii.* The y- axis indicates the fold expression levels in three developmental stages

Several scent genes responsible for floral fragrance showed significant differences among the three floral developmental stages. To confirm the sequencing results, 6 genes involved in flower fragrance biosynthesis metabolism were selected for qRT-PCR analysis. The expression patterns of these genes for each developmental stage are shown in Fig. 9. FDPS, AACT2, HMGR2–2, DXR, DXS3, and HDR, fragrance genes are expressed in the three flower developmental stages. Thus, data generated here can be used to investigate candidate flowering genes and showed comprehensive expression levels among three developmental stages. Most of the genes highly expressed in half flowering stage rather than full flowering stage and closed bud. But DXS3 showed highest expression levels in full flowering stsge.

## Discussion

### Transcriptome sequencing of *C. goeringii* flowers

*C. goeringii* is an endangered, fragrant and economically important plant primarily found in Asian countries. Floral scent components have been widely used in perfumes, cosmetics, flavorings and medicinal substances [33]. However, little information is known about the pathways responsible for floral scent. Main aim of this study was to produce a large amount of cDNA sequence data for more detailed studies of *C*. *goeringii* and to identify genes involved in the synthesis of floral scent compounds. Specifically, we are interested in scent compound synthesis and emission peaks at advanced stages of flower development (between cell expansions). Present availability of *C*. *goeringii* transcriptome data provides resource for further functional investigations in this species and its relatives. The Illumina based RNA-Seq data generated 159,616,374 clean reads that were assembled into 85,868 final unigenes, with an average sequence length of 1194 nt. The average length of the final unigenes was greater than that obtained for other plants, such as *Hedychium coronarium* (732 bp) [14], *S*. *oblata* (853 bp) [18], *C*. *sinense* (612 bp) [20], *Myrica rubra* (437 bp) [34], bamboo (736 bp) [35], and *Hevea brasiliensis* (485 bp) [36], by using similar sequencing technologies. These annotated against the NCBI nr protein databases, including SwissProt, KOG, KEGG, and GO. Among all the unigenes, 56,808 (66.15%) were identified through BLAST searches, while 33.85% of unigenes showed no similarities in the NCBI database. This suggests that the vegetative and reproductive stages of *C*. *goeringii* may involve many unique processes and pathways. However, flowers are still the major resource for the production of scent compounds.

### Volatile terpenoid metabolism genes in *C. goeringii*

Terpenoids are the most important components in the *C*. *goeringii* floral scents. Emission of terpenes and floral scent biosynthesis has been studied in many plants, including *S. oblata* [18] [18], *Clarkia breweri* [37, 38], *A. thaliana* [39] and *Lavandula angustifolia* [40]. In *C*. *goeringii*, floral scent genes involved in the terpenoid pathways. We identified terpenoid pathway genes including farnesyl diphosphate synthase (FDPS), acetyl-CoA C-acetyltransferase (AACT), hydroxy-3-methylglutaryl coenzyme A reductase (HMGR), (E)-4-hydroxy-3-methylbut-2-enyl- diphosphate synthase (HDS) and hydroxymethylglutaryl-CoA synthase (HMGS), which as responsible for floral scent. Moreover we were identified which as 1-deoxy-d-xylulose-5-phosphate synthase **(**DXS), 1-deoxy-d-xylulose-5-phosphate reductoisomerase (DXR). All these synthases to the biosynthesis of terpenoids in *C*. *goeringii*. Since farnesol is the main compound in the investigated cultivar, this study four genes FDPS, HMGS, HMGR, and AACT in the MVP pathway and analyzed their expression at flowering stages. The results support that farnesol is synthesized through the MVP as shown in Fig. 4, and its biosynthesis and emission are closely correlated with to the expression levels of those genes. They showed similar expression patterns among the three flower developmental stages. They showed the highest expression at stage C (Fig. 8) (the full flowering stage), at which the emission of volatile terpenoids has been shown to be high. In *S. oblata*, terpenoid biosynthesis genes involved in the MVA and MEP pathways had been previously identified: DXS, DXR, HMGR, GPS, TPS3, TPS, and LIS [18]. These genes regulated among various development stages and had the strongest expression during the full flowering stage. Farnesyl pyrophosphate synthase (FPPS) catalyzes the biosynthesis of FPP, which is the precursor of floral scent volatiles such as sesquiterpenoids. Plants with high expression levels of FPPS in flowers include *Withania somnifera* [41], and *C. praecox* [42]. *Chimonanthus praecox* (wintersweet) FPPS and volatile sesquiterpenoids level analyzed in *C. praecox* flowers reveal that FPPS may play a regulatory role in the sesquiterpenoid pathway in this species. Expression studies of two kiwi fruit (*Actinidia deliciosa*) synthases, farnesene synthase (AdAFS1) and germacrene synthase (*AdGDS1*), showed that expression of these genes was significantly higher in flowers than in leaf tissue [43]. Within floral tissues, expression of both genes was highest in petals and stamens [44]. The DXS and DXR genes isolated from *R. rugosa* flowers also show consistent expression during development, from budding to the withering stage [8]. In *S. oblata* [18], the expression of the DXS and DXR genes is positively correlated with the emission of volatile terpenoids during full blooming of the inflorescence stage. In *rose*, high levels of DXS and DXR expression were also found in flowers, consistent with the relatively high emission of terpenoids from this part of the plant [45]. GDPS, a gene that participates in the biosynthesis of monoterpenes in plastids [46], has also been shown to be differentially expressed among different flower developmental stages in *P. bellina* and *P. equestris* [47, 48]. Benzenoids related genes S-adenosyl-L-methionine-dependent methyltransferases and O-methyltransferase were expressed at full flowering stage and partial flowering stages. *O*-methyltransferases were shown to be responsible for the synthesis of a diverse array of benzenoids/ phenylpropanoids, including veratrole in *Silene* flowers. S-adenosyl-L-methionine: salicylic acid carboxyl methyltransferase, and theobromine synthase (SABATH) family are involved in the biosynthesis of volatile esters like methylbenzoate in snapdragon and petunia flowers.

### Transcription factors involved in floral scent synthesis in *C. goeringii*

Transcription factors control gene expression and play an important role in a number of biological pathways in plants. However, very little is known about the regulation of floral scent production at the molecular level. Analysis of transcription factor expression levels in *C*. *goeringii* is necessary to understand their role in the biosynthesis of secondary metabolites. Previous studies have shown that MYB and ERF play an important role in terpenoid metabolism [18]. ODORANT1 (ODO1) was the first transcription factor identified as a regulator of scent production in flowers [41]. EMISSION OF BENZENOIDS I and II (EOBI and EOBII), two genes belonging to the R2R3-MYB family (subgroup 19), have also been recently shown to regulate benzenoid biosynthesis pathways in petunias [48-50]. *EOBI* and *EOBII* positively regulate *ODO1*, which has been shown to regulate floral scent production in *P*. *hybrida*. However, the mechanism by which transcription factors regulate terpene biosynthesis has not yet been elucidated. In the present study, we isolated candidate TFs for regulating terpene biosynthesis, by analyzing the expression pattern of the structural genes encoding putative enzymes involved in terpene biosynthesis *C*. *goeringii*. Intriguingly, we found that the known TFs for regulating terpene biosynthesis, including ERF, NAC, MYB, and bHLH, occupied a large proportion in these DEGs.

## Conclusions

Using the Illumina RNA- sequencing and DEGs analysis based results produced compressive information on gene expression levels in *C*. *goeringii.* According to these data, we identified numerous differentially expressed genes in three flower developmental stages. Genes responsible for terpenoids were readily identified in stage2. The present data could be used as a tool to investigate further flowering scent biological pathways in *C*. *goeringii* and also helps to improve the horticultural and other economically important ornamental plants through floral scents in the species.

## Methods

### Plant materials

*Cymbidium goeringii* plants were collected from an orchid farm in Puli, Nan-Tou, at central Taiwan. Various floral developmental stages of *C*. *goeringii* were selected for the RNA- seq analysis, including the floral bud (stage A), the half flowering stage (stage B) and the full flowering stage (stage C) (Fig. 2). Plant samples from each stage were collected from three plants and frozen immediately in liquid nitrogen and kept at − 80 °C for further analysis.

### GC-MS analysis

The floral volatiles were analyzed using HS-SPME-GC-MS which is 7000C GC-MS system (Agilent technologies, Wilmington, DE, USA). The GC was equipped with a DB-5MS column (30 m⨯0.25 mm I.D.⨯0.25 μm, Agilent Technologies, Wilmington, DE, USA). With its temperature held at 60 °C for 5 min and then raised to 250 °C at 3 °C /min. The injector and detector temperature was maintained at 250 °C. The carrier gas helium flow rate was 1.0 mL/min. The MS detector was used in the EI mode with an electron energy at 70 eV, and by full scanning the data at a rate of 1 scan/s over the m/z range of 30–350 amu. The transfer line was at 280 °C. The identification of HS-SPME-GC-MS was performed by comparison with n-alkane and NIST 13 (National Institute of Standards and Technology, Gaithersburg, MD, USA) mass spectral library, and retention indices (RI) of the compounds determined by using Kovat index. The GC-MS data of volatile compounds values were shown by means ± SD of triplicates. The SPPS program (SPPS Inc., Chicago, IL, USA) was used for the distribution of volatile constituent. Duncan’s multiple range test was carried out to check the volatile emission changes of main compounds during day cycle.

### RNA extraction

*C*. *goeringii* floral samples were ground into powder in a mortar with liquid nitrogen, and total RNA was extracted from three samples of developmental stages using Trizol (Invitrogen, USA) according to the manufacturer’s protocol. Quality and quantity of RNA were checked using a spectrophotometer (Agilent 2100 UV visible spectrophotometer, Santa Clara, Canada) and analyzed in a 1% agarose gel. The transcriptome sequencing library prepared by mixing of equal quantities of RNA from the three plants (for three developmental stages).

### Construction of cDNA library for Illumina sequencing

For transcriptome sequencing, cDNA libraries were prepared from RNA of three different floral developmental stages. Briefly, poly (A) mRNA was isolated from the RNA using Oligo (T) magnetic beads. cDNA was synthesized using the mRNA fragments as templates. The resulting short cDNA fragments were purified with a QIAquick PCR extraction kit and resolved in EB buffer (TaKaRa kit). Then, sequencing was performed by Illumina HiSeq™2000 platform (BGI) and 100 bp paired-end format raw reads were generated according to the manufacturer‘s instructions. Short fragments were purified with the QIAquick PCR purification (Qiagen) extraction kit and then resolved with elution buffer for end-repair and the addition of poly (A). After the fragment ends were repaired and the poly (A) was tailed, the short fragments were ligated to sequencing adapters. Suitable fragments were selected as templates for PCR amplification, and then separated by agarose gel electrophoresis. Finally, the sequencing library was produced by PCR amplification and sequenced using the HiSeq™ 2000 platform (Illumina) at the Beijing Genomics Institute (BGI).

### Sequence data analysis and de novo assembly

The resulting low quality raw sequencing reads with low quality (for less than 20 bp) were filtered out, and so were count reads with an N percentage (percentage of nucleotides in a read that could not be sequenced) > 5% and reads containing > 20% nucleotides with a Q-value ≤10. The Q-value represents the sequencing quality of related nucleotides. The clean reads were then assembled using the Trinity software (release-20130225) [51]. Trinity first combines reads with a certain overlap length to form longer fragments, which are contigs. The reads are then mapped back to the contigs. Finally, Trinity connects the contigs and gets sequences that cannot be extended on either end. Unigenes from each assembly can then be used for further processing (e.g., sequence splicing and redundancy removal) with a sequence clustering software. After this processing, non-redundant unigenes are identified and these are then used for functional annotations.

### Sequence annotation and classification

For functional annotations, all assembled unigenes were employed for homology search against NR (NCBI non-redundant) database using an E-value cut-off of 10^− 5^. After sequence assembly, the unigenes were aligned using BLASTX to protein databases such as Swiss-Prot, the Kyoto Encyclopedia of Genes and Genomes (KEGG), the Clusters of Orthologus Groups (COG) and the Gene Ontology (GO), and the best alignment results were used for unigene sequence direction determination. For other sequences not involved in the BLAST searches, we used the ESTScan program (version 3.0.2, http://www.ch.embnet.org/software/ESTScan2.html) to predict coding sequence (CDS) and orientation. Following NR annotation, the Blast2GO program version 2.5.0 (https://www.blast2go.com/) was used to classify unigenes based on GO terms [52]. After GO classification, the WEGO software [53] was used to perform GO function classification for all unigenes and to analyze the distribution of *C. goeringii* gene functions at the macro level. Using the KEGG pathway database and NR annotation on KEGG, we were able to assign multiple unigenes to the same GO terms and the same KEGG pathway [54, 55].

### Expression analysis

*C*. *goeringii* final unigenes differential expressions among three developmental stages were studied using the edgeR software [23, 24]. Gene expression differences were evaluated using a chi-square test and the false discovery rate (FDR) was also controlled. Genes that had an FDR < 0.001 and for which the FPKM estimate was 2-fold higher than that of the lowest one were identified as differentially expressed genes (DEGs). GO enrichment annotations of DEGs were calculated using the GO:TermFinder software (version v0.86). We used a corrected *P*-value ≤0.05 or a Q-value ≤0.05 as a threshold for “enriched” DEGs. The Pathfinder Internal software was used for analysis of statistical enrichment of DEGs in KEGG pathways [54, 55]. A heat map was generated to describe the significantly altered genes during the three stages. The raw intensity data (FPKM) were log2 transformed and then used for the Z scores calculation.

### Quantitative real-time PCR (q RT-PCR)

Total RNA from three different floral stages was isolated using the Qiagen RNA plant mini kit with one column DNAse digestion (Qiagen). A total of 200 ng of RNA was used for reverse transcription with dT18 primers, and 1 μL of this reverse transcription product diluted to 20 μL of ddH2O was used as a template, using the Primescript RT reagent kit with a gDNA eraser (TaKaRa). The cDNA diluted to 200 ng/μL was used for a qPCR assay on the Rotar-Gene 6000 real-time rotary analyzer system. q-RT-PCR was performed using the SYBR Premix Ex Taq Kit (TaKaRa) according to the manufacturer’s protocol. The Actin gene was used as an internal control. Three replications of each sample were used for q-RT-PCR analysis. Values are evaluated as the means ± standard deviation.

## Additional files

Additional file 1: **Figure S1.** The length distribution of unigenes in the floral transcriptome of *C. goeringii* according to their size. (DOCX 318 kb)

Additional file 2: **Figure S2.** BLAST result analysis of the *C. goeringii* floral transcriptome against the NR database. (DOCX 221 kb)

Additional file 3: **Table S1.** The unigenes annotated as putative enzymes involved in the MVA pathway. (DOCX 18 kb)

Additional file 4: **Table S2.** The unigenes annotated as putative enzymes in the MEP pathway. (DOCX 18 kb)

Additional file 5: **Table S3.** The unigenes annotated as putative terpene synthases classifed in TPS-a clade. (DOCX 17 kb)

Additional file 6: **Table S4.** KEGG enrichment pathway analysis of DEGs. (DOCX 25 kb)

Additional file 7: **Figure S3.** Family distribution of putative transcription factors in the *C. goeringii* floral transcriptome. (DOCX 284 kb)

Additional file 8: **Table S5.** TFs isolated as the candidates regulating terpenoids biosynthesis. (DOCX 22 kb)

## Data Availability

The datasets supporting he results of this article are included with in the manuscript and the additional files.

## Abbreviations

AACT: Acetoacetyl-CoA transferase
DEGs: Differentially expressed genes
DMAPP: Dimethylallyl diphosphate
DXR: 1-deoxy-dxylulose-5-phosphate reductoisomerase
DXS: Deoxy-d-xylulose-5-phosphate synthase
FPKM: Fragments per kilobase per million reads
FPP: Farnesyl diphosphate
GGPP: Geranyl geranyl diphosphate
GPP: Geranyl diphosphate
HDR: Hydroxy-2-methyl-2-(E)-butenyl-4-diphosphate reductase
HDS: Hydroxy-3-methylbut-2-enyl-diphosphate
HMGR: Hydroxymethylglutaryl-CoA reductase
HMGS: Hydroxymethylglutaryl-CoA synthase
IPP: Isopentenyl diphosphate
LIS: Linalool synthase
MCT: Methyl-D-erythritol 4-phosphate cytidylyltransferase
MDS: Methyl-D-erythritol 2,4-cyclodiphosphate synthase
MEP: Methylerythritol phosphate
MVA: Mevalonic-acid
MVD: Mevalonate decarboxylase
MVK: Mevalonate kinase
PMK: Phosphomevalonate kinase
qRT-PCR: Quantitative reverse transcription PCR
STPs: Sesquiterpene synthases
TPS: Terpene synthase
