# Supplementary material for: RNA sequencing analysis of Cymbidium goeringii identifies floral scent biosynthesis related genes
Source: BMC Plant Biol. 2019 Aug 2;19:337. doi: 10.1186/s12870-019-1940-6 (PMC6679452; doi:10.1186/s12870-019-1940-6)
Supplement: Supplementary file 1 — Figure S1. The length distribution of unigenes in the floral transcriptome of C. goeringii according to their size. (DOCX 318 kb) [file 12870_2019_1940_MOESM1_ESM.docx]

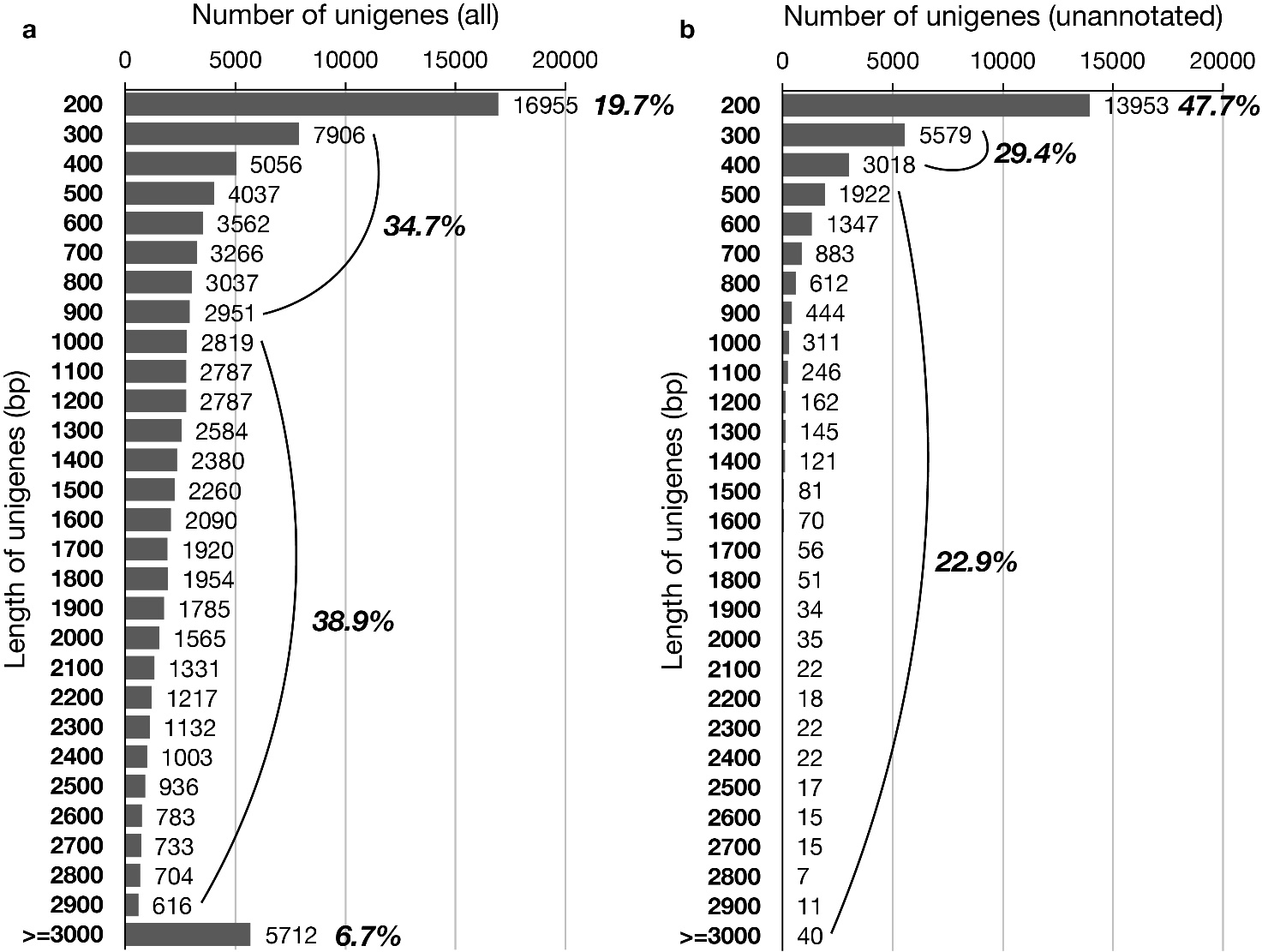


**Additional file 1: Figure S1** **The length distribution of unigenes in the floral transcriptome of *C. goeringii* according to their size.** **(a)** The length distribution of all unigenes. **(b)** The length distribution of all annotated unigenes.The number on the y-axis indicates the length range of the unigenes, for example, 200 indicates the unigenes with the length between 200- to 299-bp, 300 indicates the unigenes with the length between 300- to 399-bp, and so on. The actual number of the unigenes for each length range is shown next to the column. The curve indicates the proportion of the unigenes within the size range.
