# Supplementary material for: RNA sequencing analysis of Cymbidium goeringii identifies floral scent biosynthesis related genes
Source: BMC Plant Biol. 2019 Aug 2;19:337. doi: 10.1186/s12870-019-1940-6 (PMC6679452; doi:10.1186/s12870-019-1940-6)
Supplement: Supplementary file 2 — Figure S2. BLAST result analysis of the C. goeringii floral transcriptome against the NR database. (DOCX 221 kb) [file 12870_2019_1940_MOESM2_ESM.docx]

**
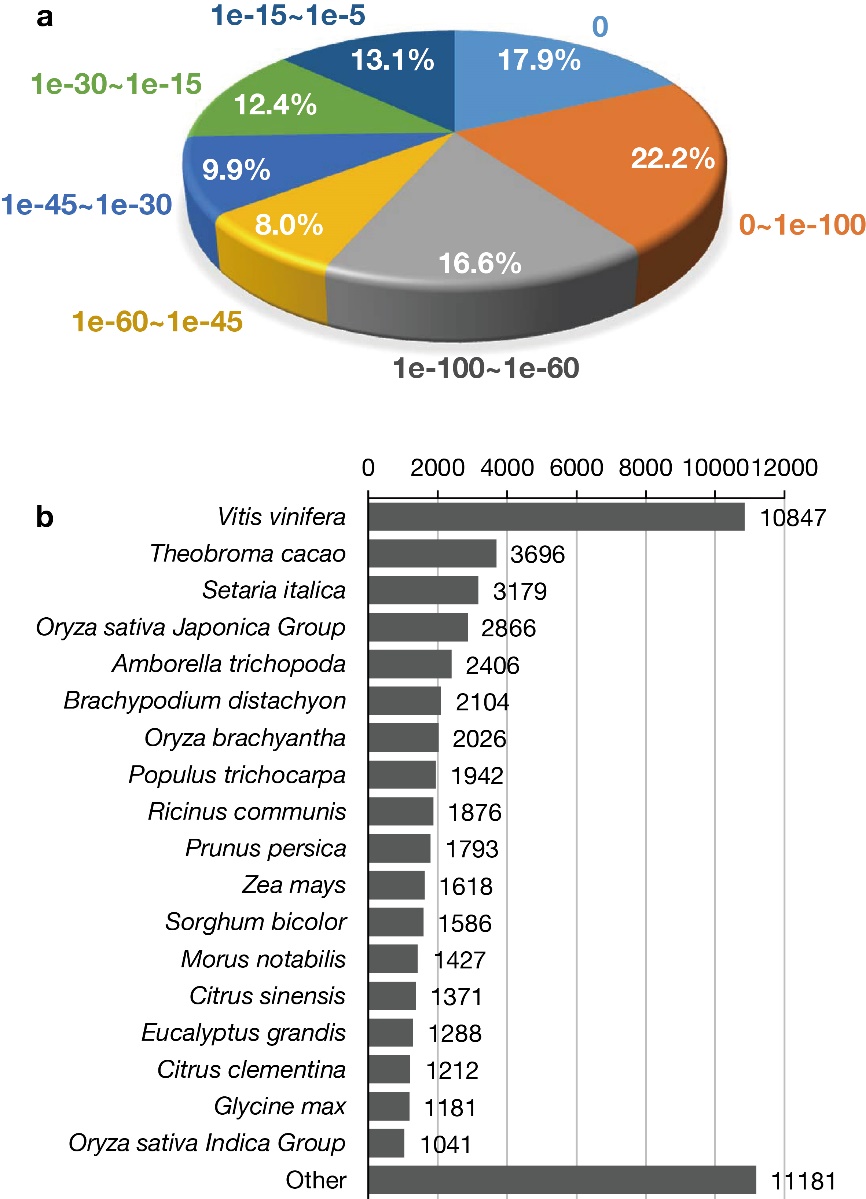
Additional file 2: Figure S2 BLAST result analysis of the *C. goeringii* floral transcriptome against the NR database.** **(a)** The E-value distribution. **(b)** The top 20 species contributing to the annotation results.
