# Supplementary material for: RNA sequencing analysis of Cymbidium goeringii identifies floral scent biosynthesis related genes
Source: BMC Plant Biol. 2019 Aug 2;19:337. doi: 10.1186/s12870-019-1940-6 (PMC6679452; doi:10.1186/s12870-019-1940-6)
Supplement: Supplementary file 3 — Table S1. The unigenes annotated as putative enzymes involved in the MVA pathway. (DOCX 18 kb) [file 12870_2019_1940_MOESM3_ESM.docx]

| **Additional file 3: Table S1 The unigenes annotated as putative enzymes involved in the MVA pathway.** | | | | | | |
| --- | --- | --- | --- | --- | --- | --- |
|  |  | KEGG annotation | | FPKM values | | |
| Transcriptome ID | Name | Evalue | Subject | A | B | C |
| CL3189.Contig4_All | CgAACT1 | 0 | acetyl-CoA C-acetyltransferase [EC:2.3.1.9] | 5.67 | 12.93 | 13.56 |
| Unigene10608_All | CgAACT2 | 0 | acetyl-CoA C-acetyltransferase [EC:2.3.1.9] | 7.38 | 23.09 | 16.64 |
| Unigene15934_All | CgAACT3 | 0 | acetyl-CoA C-acetyltransferase [EC:2.3.1.9] | 3.55 | 7.51 | 6.72 |
| Unigene18775_All | CgAACT4 | 0 | acetyl-CoA C-acetyltransferase [EC:2.3.1.9] | 1.44 | 2.46 | 3.11 |
| Unigene19278_All | CgAACT5 | 0 | acetyl-CoA C-acetyltransferase [EC:2.3.1.9] | 4.57 | 7.97 | 7.82 |
| Unigene27609_All | CgAACT6 | 0 | acetyl-CoA C-acetyltransferase [EC:2.3.1.9] | 1.86 | 2.90 | 3.08 |
| Unigene27610_All | CgAACT7 | 0 | acetyl-CoA C-acetyltransferase [EC:2.3.1.9] | 6.29 | 12.62 | 13.11 |
| Unigene9715_All | CgHMGS | 0 | hydroxymethylglutaryl-CoA synthase [EC:2.3.3.10] | 351.20 | 889.09 | 573.38 |
| CL1997.Contig2_All | CgHMGR1-1 | 0 | hydroxymethylglutaryl-CoA reductase (NADPH) [EC:1.1.1.34] | 2.21 | 181.99 | 306.87 |
| CL1997.Contig3_All | CgHMGR1-2 | 0 | hydroxymethylglutaryl-CoA reductase (NADPH) [EC:1.1.1.34] | 1.26 | 68.68 | 95.90 |
| CL4756.Contig1_All | CgHMGR2-1 | 0 | hydroxymethylglutaryl-CoA reductase (NADPH) [EC:1.1.1.34] | 4.45 | 6.79 | 5.64 |
| CL4756.Contig2_All | CgHMGR2-2 | 0 | hydroxymethylglutaryl-CoA reductase (NADPH) [EC:1.1.1.34] | 1.60 | 3.09 | 1.96 |
| CL969.Contig1_All | CgMVK-1 | 6E-137 | mevalonate kinase [EC:2.7.1.36] | 3.20 | 4.79 | 2.41 |
| CL969.Contig2_All | CgMVK-2 | 1E-64 | mevalonate kinase [EC:2.7.1.36] | 0.35 | 0.45 | 0.18 |
| CL969.Contig3_All | CgMVK-3 | 1E-64 | mevalonate kinase [EC:2.7.1.36] | 1.17 | 1.67 | 0.78 |
| CL969.Contig4_All | CgMVK-4 | 5E-137 | mevalonate kinase [EC:2.7.1.36] | 0.33 | 0.76 | 0.48 |
| CL3117.Contig1_All | CgPMK-1 | 2E-87 | phosphomevalonate kinase [EC:2.7.4.2] | 3.54 | 2.86 | 3.23 |
| CL3117.Contig2_All | CgPMK-2 | 0 | phosphomevalonate kinase [EC:2.7.4.2] | 3.05 | 2.20 | 2.92 |
| CL3117.Contig3_All | CgPMK-3 | 3E-87 | phosphomevalonate kinase [EC:2.7.4.2] | 1.08 | 0.87 | 1.02 |
| CL3117.Contig4_All | CgPMK-4 | 3E-174 | phosphomevalonate kinase [EC:2.7.4.2] | 5.25 | 2.70 | 3.83 |
| CL3117.Contig5_All | CgPMK-5 | 2E-174 | phosphomevalonate kinase [EC:2.7.4.2] | 13.71 | 12.58 | 15.83 |
| CL3117.Contig6_All | CgPMK-6 | 0 | phosphomevalonate kinase [EC:2.7.4.2] | 0.87 | 0.70 | 0.89 |
| CL3117.Contig7_All | CgPMK-7 | 6E-87 | phosphomevalonate kinase [EC:2.7.4.2] | 5.90 | 2.47 | 4.47 |
| CL3117.Contig8_All | CgPMK-8 | 4E-174 | phosphomevalonate kinase [EC:2.7.4.2] | 4.57 | 1.58 | 2.67 |
| CL3117.Contig9_All | CgPMK-9 | 3E-87 | phosphomevalonate kinase [EC:2.7.4.2] | 1.95 | 1.11 | 1.05 |
| CL3117.Contig10_All | CgPMK-10 | 0 | phosphomevalonate kinase [EC:2.7.4.2] | 2.71 | 1.73 | 1.70 |
| CL362.Contig1_All | CgMDC-1 | 0 | diphosphomevalonate decarboxylase [EC:4.1.1.33] | 16.80 | 18.61 | 17.78 |
| CL362.Contig2_All | CgMDC-2 | 0 | diphosphomevalonate decarboxylase [EC:4.1.1.33] | 3.99 | 4.54 | 4.44 |
| CL362.Contig3_All | CgMDC-3 | 0 | diphosphomevalonate decarboxylase [EC:4.1.1.33] | 7.80 | 7.33 | 8.25 |
| Unigene19651_All | CgIDI1 | 2E-118 | isopentenyl-diphosphate delta-isomerase [EC:5.3.3.2] | 96.88 | 355.15 | 183.76 |
| Unigene6967_All | CgFDPS | 8E-160 | farnesyl diphosphate synthase [EC:2.5.1.1 2.5.1.10] | 121.63 | 749.93 | 353.27 |
