# Supplementary material for: RNA sequencing analysis of Cymbidium goeringii identifies floral scent biosynthesis related genes
Source: BMC Plant Biol. 2019 Aug 2;19:337. doi: 10.1186/s12870-019-1940-6 (PMC6679452; doi:10.1186/s12870-019-1940-6)
Supplement: Supplementary file 4 — Table S2. The unigenes annotated as putative enzymes in the MEP pathway. (DOCX 18 kb) [file 12870_2019_1940_MOESM4_ESM.docx]

| **Additional file 4: Table S2 The unigenes annotated as putative enzymes involved in the MEP pathway.** | | | | | | |
| --- | --- | --- | --- | --- | --- | --- |
|  |  | KEGG annotation | | FPKM values | | |
| Transcriptome ID | Name | Evalue | Subject | A | B | C |
| CL4095.Contig1_All | CgDXS1-1 | 0 | 1-deoxy-D-xylulose-5-phosphate synthase [EC:2.2.1.7] | 0.84 | 0.82 | 0.96 |
| CL4095.Contig2_All | CgDXS1-2 | 0 | 1-deoxy-D-xylulose-5-phosphate synthase [EC:2.2.1.7] | 0.52 | 0.44 | 0.50 |
| CL4095.Contig3_All | CgDXS1-3 | 0 | 1-deoxy-D-xylulose-5-phosphate synthase [EC:2.2.1.7] | 0.81 | 0.63 | 0.50 |
| CL4095.Contig5_All | CgDXS1-4 | 0 | 1-deoxy-D-xylulose-5-phosphate synthase [EC:2.2.1.7] | 0.93 | 0.98 | 0.88 |
| CL4095.Contig6_All | CgDXS1-5 | 0 | 1-deoxy-D-xylulose-5-phosphate synthase [EC:2.2.1.7] | 0.80 | 1.05 | 0.96 |
| Unigene1529_All | CgDXS2 | 0 | 1-deoxy-D-xylulose-5-phosphate synthase [EC:2.2.1.7] | 95.50 | 74.20 | 115.44 |
| Unigene7003_All | CgDXS3 | 0 | 1-deoxy-D-xylulose-5-phosphate synthase [EC:2.2.1.7] | 14.96 | 156.72 | 216.72 |
| Unigene6891_All | CgDXR | 0 | 1-deoxy-D-xylulose-5-phosphate reductoisomerase [EC:1.1.1.267] | 103.96 | 606.87 | 313.27 |
| CL6558.Contig1_All | CgMCT | 3E-102 | 2-C-methyl-D-erythritol 4-phosphate cytidylyltransferase [EC:2.7.7.60] | 4.36 | 2.33 | 1.84 |
| CL4350.Contig1_All | CgCMK-1 | 1E-158 | 4-diphosphocytidyl-2-C-methyl-D-erythritol kinase [EC:2.7.1.148] | 10.11 | 14.88 | 9.67 |
| CL4350.Contig2_All | CgCMK-2 | 1E-153 | 4-diphosphocytidyl-2-C-methyl-D-erythritol kinase [EC:2.7.1.148] | 4.60 | 7.13 | 3.93 |
| CL2425.Contig2_All | CgMDS-1 | 3E-88 | 2-C-methyl-D-erythritol 2,4-cyclodiphosphate synthase [EC:4.6.1.12] | 2.60 | 4.61 | 6.16 |
| CL2425.Contig3_All | CgMDS-2 | 2E-73 | 2-C-methyl-D-erythritol 2,4-cyclodiphosphate synthase [EC:4.6.1.12] | 3.62 | 6.06 | 7.36 |
| CL2425.Contig4_All | CgMDS-3 | 2E-73 | 2-C-methyl-D-erythritol 2,4-cyclodiphosphate synthase [EC:4.6.1.12] | 3.08 | 5.31 | 5.87 |
| CL2425.Contig5_All | CgMDS-4 | 4E-88 | 2-C-methyl-D-erythritol 2,4-cyclodiphosphate synthase [EC:4.6.1.12] | 5.06 | 12.50 | 12.97 |
| CL661.Contig1_All | CgHDS-1 | 0 | (E)-4-hydroxy-3-methylbut-2-enyl-diphosphate synthase [EC:1.17.7.1] | 1.86 | 4.06 | 4.51 |
| CL661.Contig2_All | CgHDS-2 | 0 | (E)-4-hydroxy-3-methylbut-2-enyl-diphosphate synthase [EC:1.17.7.1] | 1.45 | 3.06 | 3.93 |
| CL661.Contig3_All | CgHDS-3 | 0 | (E)-4-hydroxy-3-methylbut-2-enyl-diphosphate synthase [EC:1.17.7.1] | 0.78 | 1.97 | 2.25 |
| CL661.Contig4_All | CgHDS-4 | 0 | (E)-4-hydroxy-3-methylbut-2-enyl-diphosphate synthase [EC:1.17.7.1] | 5.76 | 13.20 | 13.55 |
| CL661.Contig5_All | CgHDS-5 | 0 | (E)-4-hydroxy-3-methylbut-2-enyl-diphosphate synthase [EC:1.17.7.1] | 0.99 | 2.27 | 2.70 |
| CL661.Contig6_All | CgHDS-6 | 0 | (E)-4-hydroxy-3-methylbut-2-enyl-diphosphate synthase [EC:1.17.7.1] | 0.71 | 1.85 | 2.17 |
| CL661.Contig7_All | CgHDS-7 | 0 | (E)-4-hydroxy-3-methylbut-2-enyl-diphosphate synthase [EC:1.17.7.1] | 0.82 | 2.15 | 2.46 |
| CL661.Contig8_All | CgHDS-8 | 0 | (E)-4-hydroxy-3-methylbut-2-enyl-diphosphate synthase [EC:1.17.7.1] | 0.89 | 2.28 | 2.56 |
| CL661.Contig9_All | CgHDS-9 | 0 | (E)-4-hydroxy-3-methylbut-2-enyl-diphosphate synthase [EC:1.17.7.1] | 6.34 | 6.90 | 10.18 |
| Unigene15237_All | CgHDR | 0 | 4-hydroxy-3-methylbut-2-enyl diphosphate reductase [EC:1.17.1.2] | 106.17 | 818.48 | 593.30 |
| Unigene5800_All | CgIDI2 | 1E-117 | isopentenyl-diphosphate delta-isomerase [EC:5.3.3.2] | 74.45 | 420.19 | 255.95 |
| CL7614.Contig1_All | CgGDPS1-1 | 3E-144 | geranyl diphosphate synthase [EC:2.5.1.1] | 0.77 | 0.74 | 1.06 |
| CL7614.Contig2_All | CgGDPS1-2 | 3E-144 | geranyl diphosphate synthase [EC:2.5.1.1] | 0.59 | 0.60 | 0.76 |
| CL7614.Contig3_All | CgGDPS1-3 | 3E-147 | geranyl diphosphate synthase [EC:2.5.1.1] | 0.64 | 0.68 | 0.71 |
| CL7614.Contig4_All | CgGDPS1-4 | 2E-141 | geranyl diphosphate synthase [EC:2.5.1.1] | 4.26 | 6.42 | 5.70 |
| CL7614.Contig5_All | CgGDPS1-5 | 3E-147 | geranyl diphosphate synthase [EC:2.5.1.1] | 0.63 | 0.61 | 0.78 |
| Unigene16546_All | CgGDPS2 | 2E-103 | geranyl diphosphate synthase [EC:2.5.1.1] | 4.66 | 2.07 | 1.43 |
