# Supplementary material for: RNA sequencing analysis of Cymbidium goeringii identifies floral scent biosynthesis related genes
Source: BMC Plant Biol. 2019 Aug 2;19:337. doi: 10.1186/s12870-019-1940-6 (PMC6679452; doi:10.1186/s12870-019-1940-6)
Supplement: Supplementary file 5 — Table S3. The unigenes annotated as putative terpene synthases classifed in TPS-a clade. (DOCX 17 kb) [file 12870_2019_1940_MOESM5_ESM.docx]

| **Additional file 5: Table S3 The unigenes annotated as putative terpene synthases classifed in TPS-a clade.** | | | | | | |
| --- | --- | --- | --- | --- | --- | --- |
|  |  | KEGG annotation | | FPKM values | | |
| Transcriptome ID | Name | Evalue | Subject | A | B | C |
| CL2535.Contig1_All | CgTPS2-1 | 2E-41 | valencene synthase [EC:4.2.3.73] | 0.00 | 0.81 | 0.05 |
| CL2535.Contig2_All | CgTPS2-2 | 1E-45 | valencene synthase [EC:4.2.3.73] | 0.05 | 1.23 | 0.05 |
| CL2535.Contig3_All | CgTPS2-3 | 1E-45 | valencene synthase [EC:4.2.3.73] | 0.00 | 1.24 | 0.10 |
| CL2535.Contig4_All | CgTPS2-4 | 2E-41 | valencene synthase [EC:4.2.3.73] | 0.00 | 0.87 | 0.05 |
| CL2535.Contig5_All | CgTPS2-5 | 7E-76 | valencene synthase [EC:4.2.3.73] | 0.19 | 1.22 | 0.00 |
| CL2535.Contig6_All | CgTPS2-6 | 9E-53 | valencene synthase [EC:4.2.3.73] | 0.70 | 3.70 | 0.30 |
| CL2535.Contig7_All | CgTPS2-7 | 1E-54 | valencene synthase [EC:4.2.3.73] | 0.28 | 3.23 | 0.30 |
| CL2535.Contig8_All | CgTPS2-8 | 6E-71 | valencene synthase [EC:4.2.3.73] | 0.21 | 1.77 | 0.08 |
| CL2535.Contig9_All | CgTPS2-9 | 5E-71 | valencene synthase [EC:4.2.3.73] | 0.22 | 1.95 | 0.00 |
| CL2535.Contig10_All | CgTPS2-10 | 6E-59 | valencene synthase [EC:4.2.3.73] | 0.18 | 1.02 | 0.00 |
| CL2535.Contig11_All | CgTPS2-11 | 5E-67 | valencene synthase [EC:4.2.3.73] | 0.15 | 0.82 | 0.03 |
| CL2535.Contig12_All | CgTPS2-12 | 2E-71 | valencene synthase [EC:4.2.3.73] | 0.16 | 0.85 | 0.03 |
| CL2535.Contig13_All | CgTPS2-13 | 1E-75 | valencene synthase [EC:4.2.3.73] | 0.16 | 0.90 | 0.00 |
| CL2535.Contig14_All | CgTPS2-14 | 7E-59 | valencene synthase [EC:4.2.3.73] | 0.17 | 1.39 | 0.00 |
| CL2535.Contig15_All | CgTPS2-15 | 2E-71 | valencene synthase [EC:4.2.3.73] | 0.16 | 0.89 | 0.03 |
| CL2535.Contig16_All | CgTPS2-16 | 1E-75 | valencene synthase [EC:4.2.3.73] | 0.17 | 0.97 | 0.00 |
| CL2535.Contig17_All | CgTPS2-17 | 5E-67 | valencene synthase [EC:4.2.3.73] | 0.18 | 1.30 | 0.11 |
| CL2535.Contig18_All | CgTPS2-18 | 1E-61 | valencene synthase [EC:4.2.3.73] | 0.53 | 2.14 | 0.26 |
| CL4785.Contig1_All | CgTPS4-1 | ##### | valencene synthase [EC:4.2.3.73] | 0.00 | 0.03 | 0.10 |
| CL4785.Contig2_All | CgTPS4-2 | 2E-78 | valencene synthase [EC:4.2.3.73] | 0.00 | 0.03 | 0.04 |
| CL4785.Contig3_All | CgTPS4-3 | ##### | valencene synthase [EC:4.2.3.73] | 0.00 | 0.47 | 0.24 |
| CL4785.Contig4_All | CgTPS4-4 | 1E-82 | valencene synthase [EC:4.2.3.73] | 0.00 | 0.03 | 0.04 |
| CL4785.Contig5_All | CgTPS4-5 | 6E-79 | valencene synthase [EC:4.2.3.73] | 0.00 | 0.03 | 0.03 |
| CL4785.Contig6_All | CgTPS4-6 | 4E-83 | valencene synthase [EC:4.2.3.73] | 0.00 | 0.03 | 0.12 |
| CL4785.Contig7_All | CgTPS4-7 | 3E-85 | valencene synthase [EC:4.2.3.73] | 0.00 | 0.16 | 0.23 |
| CL4785.Contig8_All | CgTPS4-8 | 9E-44 | valencene synthase [EC:4.2.3.73] | 0.00 | 0.28 | 0.17 |
| CL4785.Contig9_All | CgTPS4-9 | 2E-39 | (3S)-linalool synthase [EC:4.2.3.25] | 0.00 | 0.06 | 0.07 |
| Unigene24950_All | CgTPS7 | ##### | valencene synthase [EC:4.2.3.73] | 0.45 | 11.01 | 7.16 |
| Unigene37931_All | CgTPS8 | 9E-20 | valencene synthase [EC:4.2.3.73] | 0.00 | 0.33 | 0.07 |
| Unigene38275_All | CgTPS9 | 4E-24 | valencene synthase [EC:4.2.3.73] | 0.00 | 0.67 | 0.08 |
| Unigene39476_All | CgTPS10 | 1E-41 | valencene synthase [EC:4.2.3.73] | 0.00 | 0.07 | 0.08 |
| Unigene39477_All | CgTPS11 | 9E-38 | valencene synthase [EC:4.2.3.73] | 0.00 | 0.13 | 0.00 |
