# Supplementary material for: RNA sequencing analysis of Cymbidium goeringii identifies floral scent biosynthesis related genes
Source: BMC Plant Biol. 2019 Aug 2;19:337. doi: 10.1186/s12870-019-1940-6 (PMC6679452; doi:10.1186/s12870-019-1940-6)
Supplement: Supplementary file 6 — Table S4. KEGG enrichment pathway analysis of DEGs. (DOCX 25 kb) [file 12870_2019_1940_MOESM6_ESM.docx]

| **Additional file 6: Table S4 KEGG enrichment pathway analysis of DEGs.** | | | |
| --- | --- | --- | --- |
| Pathway | A vs B | A vs C | KEGG annotation for all unigenes |
| Metabolic pathways | 2346 | 2278 | 7483 |
| Biosynthesis of secondary metabolites | 1183 | 1164 | 3197 |
| Plant-pathogen interaction | 480 | 452 | 1532 |
| RNA transport | 500 | 426 | 1485 |
| Plant hormone signal transduction | 516 | 459 | 1478 |
| Spliceosome | 338 | 229 | 1368 |
| Endocytosis | 437 | 420 | 1105 |
| Glycerophospholipid metabolism | 406 | 405 | 1070 |
| Starch and sucrose metabolism | 328 | 310 | 977 |
| Purine metabolism | 235 | 230 | 843 |
| Ether lipid metabolism | 352 | 346 | 838 |
| Pyrimidine metabolism | 213 | 204 | 831 |
| mRNA surveillance pathway | 269 | 206 | 815 |
| Protein processing in endoplasmic reticulum | 254 | 220 | 778 |
| RNA degradation | 264 | 188 | 777 |
| Ribosome biogenesis in eukaryotes | 243 | 177 | 730 |
| Ubiquitin mediated proteolysis | 140 | 123 | 647 |
| Ribosome | 203 | 239 | 538 |
| RNA polymerase | 152 | 142 | 481 |
| Homologous recombination | 95 | 86 | 470 |
| Phenylpropanoid biosynthesis | 180 | 181 | 466 |
| Pentose and glucuronate interconversions | 180 | 176 | 450 |
| ABC transporters | 140 | 115 | 417 |
| Nucleotide excision repair | 72 | 60 | 374 |
| Amino sugar and nucleotide sugar metabolism | 101 | 89 | 367 |
| Phosphatidylinositol signaling system | 58 | 48 | 336 |
| Glycolysis / Gluconeogenesis | 128 | 124 | 329 |
| Circadian rhythm - plant | 101 | 89 | 314 |
| Phagosome | 110 | 105 | 293 |
| Inositol phosphate metabolism | 49 | 45 | 291 |
| Oxidative phosphorylation | 108 | 98 | 290 |
| DNA replication | 60 | 62 | 255 |
| Peroxisome | 80 | 67 | 245 |
| Arginine and proline metabolism | 62 | 58 | 239 |
| Cyanoamino acid metabolism | 83 | 85 | 238 |
| Flavonoid biosynthesis | 88 | 100 | 236 |
| Aminoacyl-tRNA biosynthesis | 49 | 36 | 234 |
| Regulation of autophagy | 58 | 52 | 228 |
| N-Glycan biosynthesis | 26 | 16 | 227 |
| Fructose and mannose metabolism | 59 | 54 | 227 |
| Galactose metabolism | 56 | 54 | 222 |
| Pyruvate metabolism | 85 | 94 | 217 |
| Mismatch repair | 48 | 45 | 213 |
| Porphyrin and chlorophyll metabolism | 59 | 36 | 207 |
| Other glycan degradation | 47 | 50 | 206 |
| Tyrosine metabolism | 81 | 65 | 205 |
| Terpenoid backbone biosynthesis | 89 | 81 | 197 |
| Glycosylphosphatidylinositol(GPI)-anchor biosynthesis | 41 | 36 | 193 |
| Carotenoid biosynthesis | 57 | 55 | 192 |
| Glycine, serine and threonine metabolism | 60 | 54 | 186 |
| Glutathione metabolism | 66 | 47 | 185 |
| Ubiquinone and other terpenoid-quinone biosynthesis | 68 | 59 | 185 |
| Sphingolipid metabolism | 31 | 31 | 179 |
| Ascorbate and aldarate metabolism | 57 | 57 | 177 |
| Basal transcription factors | 42 | 28 | 175 |
| Glycosaminoglycan degradation | 22 | 30 | 172 |
| Diterpenoid biosynthesis | 60 | 103 | 172 |
| Phenylalanine metabolism | 80 | 76 | 171 |
| Fatty acid metabolism | 74 | 65 | 169 |
| Base excision repair | 50 | 53 | 163 |
| Stilbenoid, diarylheptanoid and gingerol biosynthesis | 80 | 81 | 162 |
| Glycerolipid metabolism | 43 | 36 | 161 |
| Valine, leucine and isoleucine degradation | 45 | 43 | 159 |
| Cysteine and methionine metabolism | 57 | 55 | 150 |
| Photosynthesis | 39 | 42 | 147 |
| Glyoxylate and dicarboxylate metabolism | 57 | 45 | 145 |
| Lysine degradation | 41 | 30 | 145 |
| Citrate cycle (TCA cycle) | 49 | 56 | 145 |
| Carbon fixation in photosynthetic organisms | 61 | 62 | 145 |
| SNARE interactions in vesicular transport | 34 | 23 | 140 |
| Protein export | 46 | 38 | 138 |
| Zeatin biosynthesis | 65 | 73 | 137 |
| Nitrogen metabolism | 50 | 47 | 137 |
| Flavone and flavonol biosynthesis | 67 | 81 | 134 |
| Alanine, aspartate and glutamate metabolism | 45 | 48 | 133 |
| Steroid biosynthesis | 39 | 39 | 130 |
| Pentose phosphate pathway | 56 | 43 | 128 |
| Pantothenate and CoA biosynthesis | 15 | 26 | 127 |
| Cutin, suberine and wax biosynthesis | 93 | 88 | 126 |
| Limonene and pinene degradation | 68 | 64 | 122 |
| alpha-Linolenic acid metabolism | 56 | 57 | 114 |
| beta-Alanine metabolism | 38 | 29 | 107 |
| Glycosphingolipid biosynthesis - ganglio series | 15 | 22 | 106 |
| One carbon pool by folate | 15 | 8 | 102 |
| Natural killer cell mediated cytotoxicity | 26 | 29 | 100 |
| Proteasome | 53 | 37 | 98 |
| Valine, leucine and isoleucine biosynthesis | 29 | 36 | 97 |
| Biosynthesis of unsaturated fatty acids | 49 | 40 | 96 |
| Propanoate metabolism | 48 | 47 | 95 |
| Fatty acid biosynthesis | 28 | 34 | 94 |
| Non-homologous end-joining | 8 | 14 | 90 |
| Fatty acid elongation | 39 | 34 | 82 |
| Tryptophan metabolism | 39 | 40 | 82 |
| Histidine metabolism | 18 | 18 | 81 |
| Phenylalanine, tyrosine and tryptophan biosynthesis | 22 | 29 | 81 |
| Sesquiterpenoid and triterpenoid biosynthesis | 44 | 29 | 75 |
| Circadian rhythm - mammal | 7 | 6 | 74 |
| Lysine biosynthesis | 26 | 19 | 74 |
| Riboflavin metabolism | 11 | 15 | 71 |
| Folate biosynthesis | 4 | 8 | 69 |
| Butanoate metabolism | 28 | 27 | 69 |
| Brassinosteroid biosynthesis | 22 | 25 | 60 |
| Monoterpenoid biosynthesis | 33 | 35 | 56 |
| Sulfur metabolism | 18 | 23 | 56 |
| Linoleic acid metabolism | 20 | 22 | 54 |
| Isoquinoline alkaloid biosynthesis | 32 | 29 | 51 |
| Tropane, piperidine and pyridine alkaloid biosynthesis | 27 | 23 | 50 |
| Selenocompound metabolism | 11 | 14 | 49 |
| Vitamin B6 metabolism | 10 | 11 | 47 |
| Arachidonic acid metabolism | 10 | 9 | 45 |
| Nicotinate and nicotinamide metabolism | 6 | 7 | 44 |
| Other types of O-glycan biosynthesis | 10 | 8 | 43 |
| Isoflavonoid biosynthesis | 20 | 19 | 36 |
| Benzoxazinoid biosynthesis | 15 | 16 | 36 |
| Glucosinolate biosynthesis | 6 | 6 | 34 |
| Thiamine metabolism | 6 | 3 | 32 |
| Sulfur relay system | 8 | 6 | 31 |
| Taurine and hypotaurine metabolism | 8 | 8 | 22 |
| Photosynthesis - antenna proteins | 16 | 16 | 21 |
| Indole alkaloid biosynthesis | 14 | 10 | 21 |
| Glycosphingolipid biosynthesis - globo series | 6 | 5 | 18 |
| Synthesis and degradation of ketone bodies | 7 | 8 | 14 |
| Biotin metabolism | 3 | 4 | 14 |
| Lipoic acid metabolism | 3 | 0 | 12 |
| C5-Branched dibasic acid metabolism | 3 | 3 | 10 |
| Anthocyanin biosynthesis | 8 | 9 | 9 |
| Betalain biosynthesis | 4 | 4 | 5 |
