# Supplementary material for: RNA sequencing analysis of Cymbidium goeringii identifies floral scent biosynthesis related genes
Source: BMC Plant Biol. 2019 Aug 2;19:337. doi: 10.1186/s12870-019-1940-6 (PMC6679452; doi:10.1186/s12870-019-1940-6)

**Additional file 7: Figure S3 Family distribution of putative transcription factors in the *C. goeringii* floral transcriptome.**


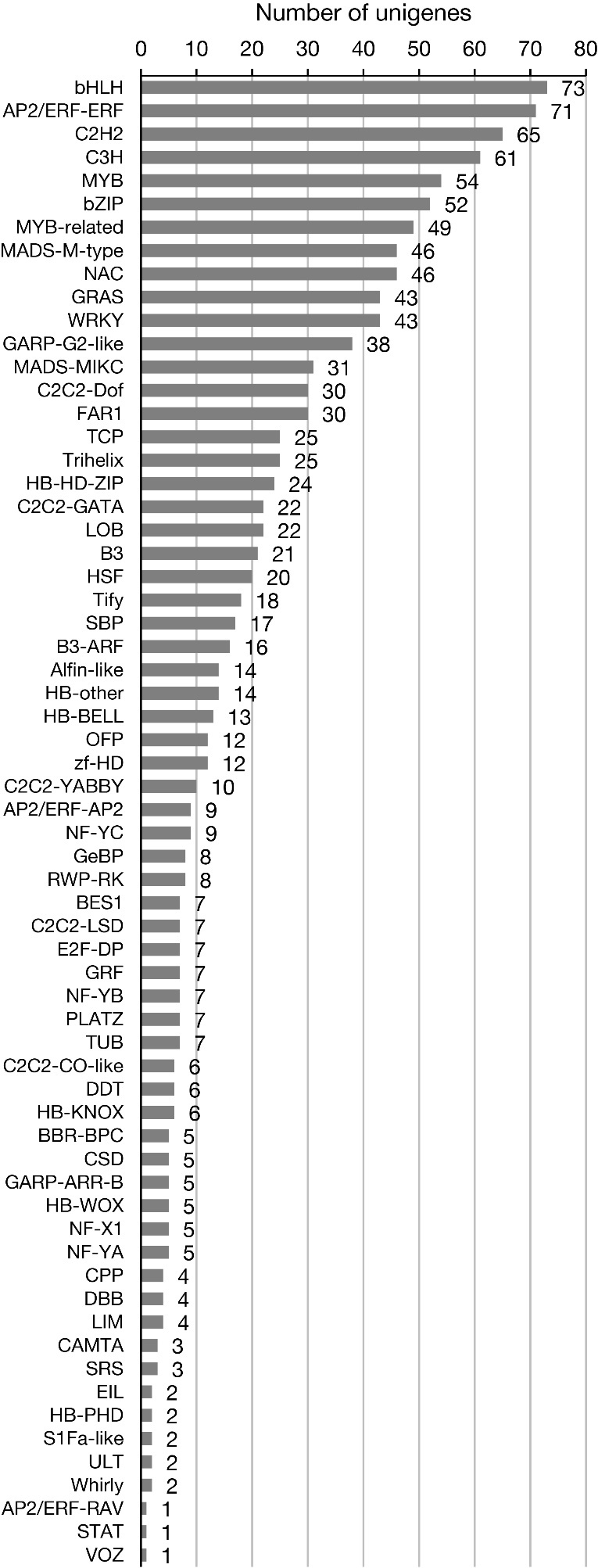

Supplement: Supplementary file 7 — Figure S3. Family distribution of putative transcription factors in the C. goeringii floral transcriptome. (DOCX 284 kb) [file 12870_2019_1940_MOESM7_ESM.docx]
