# Supplementary material for: RNA sequencing analysis of Cymbidium goeringii identifies floral scent biosynthesis related genes
Source: BMC Plant Biol. 2019 Aug 2;19:337. doi: 10.1186/s12870-019-1940-6 (PMC6679452; doi:10.1186/s12870-019-1940-6)
Supplement: Supplementary file 8 — Table S5. TFs isolated as the candidates regulating terpenoids biosynthesis. (DOCX 22 kb) [file 12870_2019_1940_MOESM8_ESM.docx]

| **Additional file 8: Table S5 TFs isolated as the candidates regulating terpenoids biosynthesis.** | | | | | | |  |  |  |
| --- | --- | --- | --- | --- | --- | --- | --- | --- | --- |
|  | Blast information to TFs regulating terpenoids biosynthesis | | | | FPKM values | | | | STEM |
| Name | Evalue | Subject | Species | Referecnes | A | B | | C | profile |
| CgbHLH1-1 | 1.8E-157 | AabHLH1 | *Artemisia annua* | Ji *et al*. 2014 | 16.19 | 27.08 | | 32.09 | 3 |
| CgbHLH1-2 | 8.3E-162 | AabHLH1 | *Artemisia annua* | Ji *et al*. 2014 | 18.63 | 18.31 | | 16.86 | 0 |
| CgbHLH1-3 | 1.8E-173 | AabHLH1 | *Artemisia annua* | Ji *et al*. 2014 | 14.20 | 28.05 | | 36.44 | 3 |
| CgbHLH2 | 2.2E-57 | CrBIS2 | *Catharanthus roseus* | van Moerkercke *et al*. 2016 | 17.66 | 4.49 | | 8.05 | 1 |
| CgbHLH3 | 0 | PbbHLH4 | *Phalaenopsis bellina* | Chuang *et al.* 2018 | 21.99 | 7.82 | | 5.47 | 0 |
| CgbZIP1 | 5.7E-134 | OsTGAP1 | *Oryza sativa* | Okada *et al.* 2009 | 0.24 | 0.20 | | 0.18 | 0 |
| CgbZIP2 | 3.7E-116 | OsbZIP79 | *Oryza sativa* | Miyamoto *et al.* 2015 | 0.28 | 0.19 | | 0.38 | 1 |
| CgbZIP3-1 | 1.1E-90 | AabZIP1 | *Artemisia annua* | Zhang *et al.* 2015 | 1.22 | 1.44 | | 1.21 | 2 |
| CgbZIP3-2 | 4.5E-90 | AabZIP1 | *Artemisia annua* | Zhang *et al.* 2015 | 3.73 | 2.65 | | 4.71 | 1 |
| CgbZIP3-3 | 1.7E-81 | AabZIP1 | *Artemisia annua* | Zhang *et al.* 2015 | 42.43 | 23.53 | | 24.99 | 0 |
| CgbZIP4 | 9.8E-117 | OsbZIP79 | *Oryza sativa* | Miyamoto *et al.* 2015 | 5.39 | 2.46 | | 3.89 | 1 |
| CgbZIP5 | 3.8E-128 | PbbZIP4 | *Phalaenopsis bellina* | Chuang *et al.* 2018 | 16.58 | 10.33 | | 8.07 | 0 |
| CgbZIP6 | 4.9E-124 | OsTGAP1 | *Oryza sativa* | Okada *et al.* 2009 | 0.39 | 0.52 | | 1.02 | 3 |
| CgbZIP7 | 1.3E-81 | PbbZIP4 | *Phalaenopsis bellina* | Chuang *et al.* 2018 | 33.08 | 40.24 | | 48.92 | 3 |
| CgbZIP8 | 1.1E-107 | PbbZIP4 | *Phalaenopsis bellina* | Chuang *et al.* 2018 | 42.84 | 37.88 | | 30.37 | 0 |
| CgERF1 | 2.3E-77 | CitAP2.10 | *Citrus sinensis* | Shen *et al.* 2016 | 4.33 | 6.45 | | 6.35 | 3 |
| CgERF2 | 5.1E-127 | CitAP2.10 | *Citrus sinensis* | Shen *et al.* 2016 | 2.30 | 31.76 | | 13.02 | 2 |
| CgERF3 | 5.2E-119 | CitAP2.10 | *Citrus sinensis* | Shen *et al.* 2016 | 7.87 | 1.76 | | 0.50 | 0 |
| CgERF4 | 2.9E-75 | CitAP2.10 | *Citrus sinensis* | Shen *et al.* 2016 | 2.93 | 3.26 | | 3.99 | 3 |
| CgMYB1 | 1.5E-51 | MsMYB | *Mentha spicata* | Reddy *et al.* 2017 | 1.58 | 4.53 | | 8.31 | 3 |
| CgMYB2 | 2.7E-104 | CrBPF1 | *Catharanthus roseus* | *Li* et al. 2015 | 0.15 | 0.02 | | 0.13 | 1 |
| CgMYB3 | 3.3E-58 | MsMYB | *Mentha spicata* | Reddy *et al.* 2017 | 29.65 | 9.96 | | 4.71 | 0 |
| CgMYB4 | 1E-50 | MsMYB | *Mentha spicata* | Reddy *et al.* 2017 | 2.32 | 0.65 | | 7.02 | 3 |
| CgMYB5 | 2.3E-61 | MsMYB | *Mentha spicata* | Reddy *et al.* 2017 | 90.04 | 7.19 | | 6.79 | 0 |
| CgNAC1 | 3.7E-79 | AaNAC2 | *Actinidia arguta* | Nieuwenhuizen *et al.* 2015 | 2.66 | 2.17 | | 21.68 | 3 |
| CgNAC2 | 5.6E-80 | AaNAC2 | *Actinidia arguta* | Nieuwenhuizen *et al.* 2015 | 4.19 | 1.32 | | 7.97 | 1 |
| CgNAC3 | 1.2E-87 | AaNAC2 | *Actinidia arguta* | Nieuwenhuizen *et al.* 2015 | 0.91 | 2.13 | | 28.82 | 3 |
| CgNAC4 | 7.3E-59 | AaNAC4 | *Actinidia arguta* | Nieuwenhuizen *et al.* 2015 | 0.66 | 0.74 | | 1.76 | 3 |
| CgNAC5 | 1.4E-75 | AaNAC4 | *Actinidia arguta* | Nieuwenhuizen *et al.* 2015 | 9.68 | 15.55 | | 28.74 | 3 |
| CgNAC6 | 2.2E-57 | AaNAC4 | *Actinidia arguta* | Nieuwenhuizen *et al.* 2015 | 3.74 | 2.31 | | 9.12 | 3 |
| CgWRKY1 | 8E-56 | GaWRKY1 | *Gossypium arboreum* | Xu *et al.* 2004 | 0.32 | 3.56 | | 13.27 | 3 |
| CgWRKY2 | 6E-59 | GaWRKY1 | *Gossypium arboreum* | Xu *et al.* 2004 | 4.67 | 4.90 | | 5.63 | 3 |
| *: CgbZIP6 was not considered as a candidate TF due to its extremely low expression levels (FPKM <1). | | | | | |  | |  |  |
